# Seed coat transcriptomic profiling of 5-593, a genotype important for genetic studies of seed coat color and patterning in common bean (*Phaseolus vulgaris* L.)

**DOI:** 10.1186/s12870-025-06282-7

**Published:** 2025-03-05

**Authors:** Jayanta Roy, Avinash Sreedasyam, Caroline Osborne, Rian Lee, Phillip E. McClean

**Affiliations:** 1 Department of Plant Sciences, North Dakota State University, Fargo, ND USA; 2 HudsonAlpha Institute for Biotechnology, Huntsville, AL USA; 3 Joint Genome Institute, Lawrence Berkeley National Laboratory, Berkeley, CA USA; 4 Genomics, Phenomics, and Bioinformatics Program, North Dakota State University, Fargo, ND USA

**Keywords:** *Phaseolus vulgaris*, Seed coat color, RNA-Seq, Flavonoids, Flavonoid biosynthesis

## Abstract

Common bean (*Phaseolus vulgaris* L.) market classes have distinct seed coat colors, which are directly related to the diverse flavonoids found in the mature seed coat. To understand and elucidate the molecular mechanisms underlying the regulation of seed coat color, RNA-Seq data was collected from the black bean 5-593 and used for a differential gene expression and enrichment analysis from four different seed coat color development stages. 5-593 carries dominant alleles for 10 of the 11 major genes that control seed coat color and expression and has historically been used to develop introgression lines used for seed coat genetic analysis. Pairwise comparison among the four stages identified 6,294 differentially expressed genes (DEGs) varying from 508 to 5,780 DEGs depending on the compared stages. Kyoto Encyclopedia of Genes and Genomes (KEGG) enrichment analysis revealed that phenylpropanoid biosynthesis, flavonoid biosynthesis, and plant hormone signal transduction comprised the principal pathways expressed during bean seed coat pigment development. Transcriptome analysis suggested that most structural genes for flavonoid biosynthesis and some potential regulatory genes were significantly differentially expressed. Further studies detected 29 DEGs as important candidate genes governing the key enzymatic flavonoid biosynthetic pathways for common bean seed coat color development. Additionally, four gene models, Pv5-593.02G016100, 593.02G078700, Pv5-593.02G090900, and Pv5-593.06G121300, encode MYB-like transcription factor family protein were identified as strong candidate regulatory genes in anthocyanin biosynthesis which could regulate the expression levels of some important structural genes in flavonoid biosynthesis pathway. These findings provide a framework to draw new insights into the molecular networks underlying common bean seed coat pigment development.

**Supplementary Information:**

The online version contains supplementary material available at 10.1186/s12870-025-06282-7.

## Introduction

Common bean (*Phaseolus vulgaris* L.) is one of the most economically important edible legumes cultivated and consumed worldwide. This crop serves as a major source of dietary protein for millions of people. It has the greatest societal impact on the smallholder farmers world because of their dependence on bean as a family food and cash crop [1]. The common bean seed is a complex organ composed of cotyledons and a well-developed embryo encircled by maternally derived seed coat. The seed coat comprises 7 to 10% of the dry matter, the cotyledon represents up to 85% or more of the dry matter, and the embryo is only 2 to 3% of the seed weight [2]. The seed coat is vital for embryo protection from mechanical damage, pathogen attack, seed desiccation, and promotion of seed dispersal. The seeds of the many bean market classes vary phenotypically in size, shape, and seed coat color and pattern. Individual market classes are regionally preferred by consumers throughout the world, and strict adherence to the phenotype of each new variety within a market class influences their market acceptance [3].

The genetic background [4–8] and distribution of pigments [9] associated with the phenotypic variation of bean seed coat color and pattern has been studied over the last several decades. An extensive network of genes (*P*, *C*, *R*, *J*, *G*, *B*, *V*, *Rk*, *T*, *Z*, and *Bip*) control color and patterning of bean seeds [6, 8]. In a biochemical context, the expression of color in the seed coat is attributed to the presence and concentration of flavonoid in the seed coat [1, 10, 11]. Flavonols, flavan-3-ols, anthocyanidins, and proanthocyanidins are the primary flavonoids found in the seed coats of beans. Flavonoids are found in many market classes of beans and positively affect human health as anti-oxidant, anti-obesity, anti-diabetic, and anti-mutagenic agents [12–14]. Of the major market classes grown in the US, anthocyanins are the major class of flavonoids in black beans, while flavonols are the major class of flavonoids in pinto, pink, medium-sized Durango red, and dark and light red kidney beans [15–16]. The study of the genetics of seed coat color and pattern, and flavonoid composition has benefited significantly from the development of introgression lines in which a single or multiple classic seed coat color and/or pattern genes were introduced into the common black bean 5-593, a Florida dry bean breeding genotype developed by Dr. Mark J. Bassett. 5-593 (genotype: *T Z Bip P* [*C r*] *J G B V Rk Gy sal*) carries the dominant allele for the seed coat color and pattern for all but two of the genes [17]. So first understanding the expression of the genes in the seed coat of 5-593 will provide a background to study the molecular effect of individual genes in the introgression lines.

**Fig. 1:**
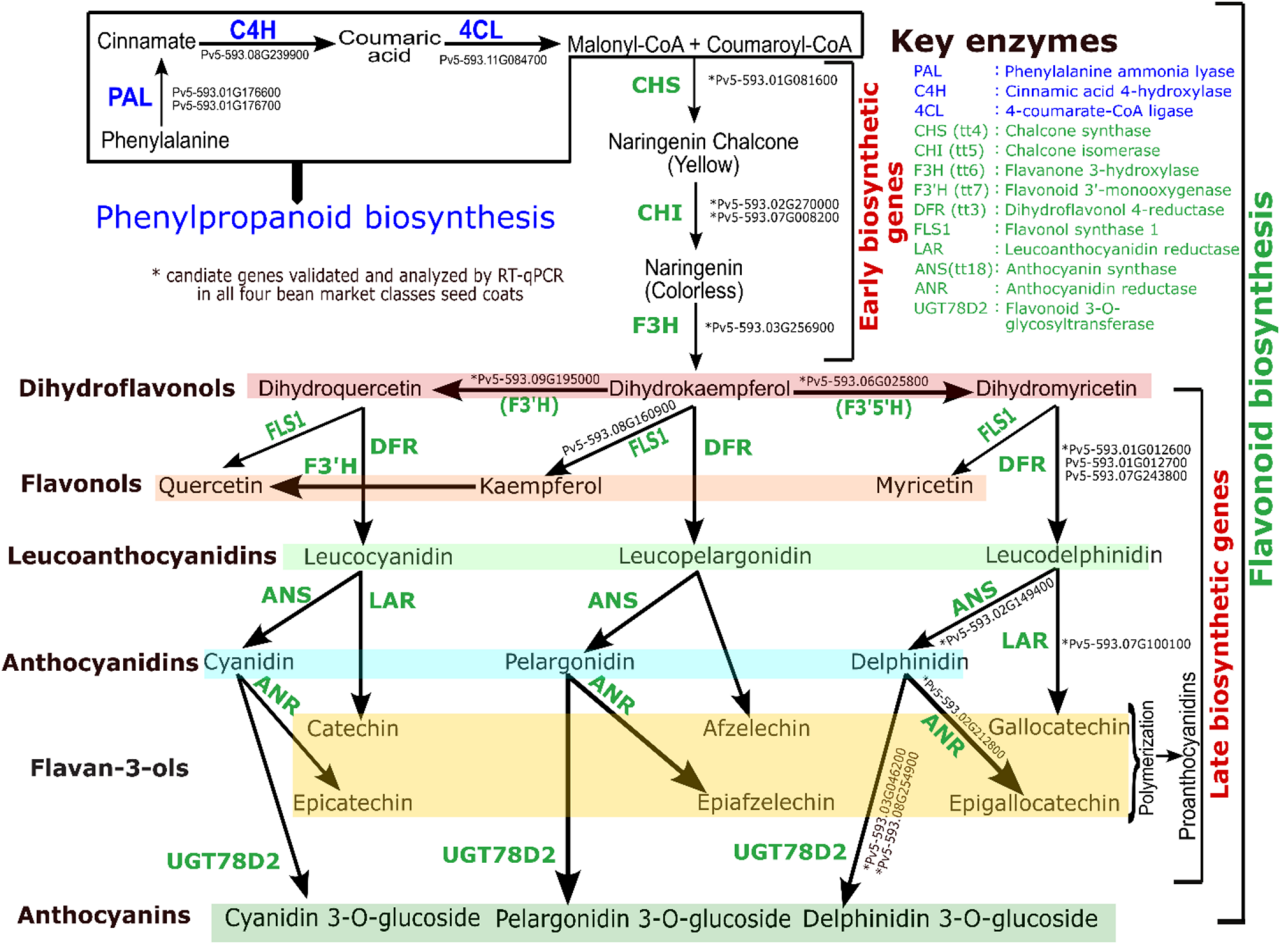
Flavonoid biosynthesis pathway in plants. The 5-593 candidate genes governing the key enzymes associated with each of the steps were provided along with each involved process

The molecular biology and biochemistry of flavonoid production is well established in model plants. The initial substrates for the flavonoid pathway are malonyl-CoA and coumaroyl-CoA from the phenylpropanoid (Fig. 1). The pathway includes early biosynthetic genes (EBGs) [EBG: naringenin-chalcone synthase/Flavanone synthase (*CHS*), chalcone isomerase (*CHI*), naringenin 3-dioxygenase (*F3H*), flavonol synthase 1 (*FLS*)] involved in the synthesis of flavnols and common precursors for the downstream pathway. These downstream products, anthocyanins, flavan-3-ols, and proanthocyanidins are generated by the late biosynthetic genes (LBGs) [LBG: bifunctional dihydroflavonol 4-reductase/flavanone 4-reductase (*DFR*), leucoanthocyanidin dioxygenase (*ANS*), leucoanthocyanidin reductase (*LAR*), anthocyanidin reductase (*ANR*)]. In addition, glycosyl transferase enzymes add sugar moieties to the basic flavonol and anthocyanidin structures. The regulation of the genes encoding these enzymes is also conserved. MYB transcription factors typically regulate the EBGs, while the LBGs required for anthocyanin and proanthocyanin biosynthesis are controlled by a MBW protein complex consisting of MYB and bHLH transcription factors that are anchored by a WD40 protein [18].

The flavonoid biosynthesis pathway and its regulatory genes are involved in determining seed coat color and the accumulation of proanthocyanidins. Two genes involved in seed coat color in common bean have been cloned. The *P* (Pigment), a master regulator gene of the flavonoid pathway, an ortholog of the Arabidopsis *TT8* gene, encodes a bHLH protein forming a component of the MBW regulatory complex. The dominant *P* allele is required for the expression of any color in the seed coat [19]. Three genes, *G*, *B*, and *V* are color-modifying genes and interact in various allelic combinations to color the seed from yellow to black [12, 20]. The *V* (“violet factor”) gene is involved in the production of color ranging from pale violet to black depending upon which *G* and *B* alleles are present. *V* maps to chromosome Pv06 and encodes flavonoid 3′5′ hydroxylase (F3′5′H; Pv5-593.06G025800), a P450 enzyme required for the expression of dihydromyricetin-derived flavonoids in the flavonoid pathway [1]. *B* (“greenish-brown factor”) maps to chromosome Pv02, and the dominant allele is required for dark violet and black seed coat colors, again depending on the allelic state of the *G* and *V* alleles. *B* regulates the amount of anthocyanins production, and seed coats of lines recessive for *b* expressed only 19% of the anthocyanin content than dominant *B* lines [21]. The *G* gene (“yellow-brown factor”) controls the yellow-brown seed coat color and maps to Pv04. An additional color gene, *Gy*, mapped on Pv08, controls the intensity of the green color [22]. The genes, *J*, *T*, *Z*, *Bip*, and genes within the complex *C* locus, are involved in the expression of seed color patterns in the partly colored seed coats [22–25]. The *T* and *Z* candidate genes were recently identified, encoding WDR and MYB proteins, respectively [24]. Computational modeling demonstrated that these proteins interact with the P protein in a domain-specific manner to form the MBW complex. *T* is homologous to the Arabidopsis WDR-encoding gene *TTG1*, while *Z* is homologous to the Arabidopsis MYB-encoding gene *TT2*. Further, recessive *P*, *T*, and *Z* alleles altered the predicted MBW structure in a manner that would compromise the function of the complex.

The accumulation of flavonols, anthocyanins, flavan-3-ols requires the coordinated expression of many pathways and regulatory genes. Moreover, it can be hypothesized that the expression of any individual gene varies at different stages of seed coat color development. Expression dynamics of flavonoid pathway genes within the seed coat have not been previously studied. While gene expression has been evaluated in whole grain [26], but the results did not differentiate between gene expression in the seed coat and that of the cotyledon and embryo. This study specifically investigates the dynamic expression patterns of flavonoid pathway genes during seed coat development in the black bean genotype 5-593. This genotype is especially relevant because it has been used for extensive studies in seed color and patterning in common bean [6]. Understanding the expression in this genotype that carries dominant alleles for the genetically defined major color and regulatory genes will provide a basis to compare other market classes that carry defined recessive genes. A RNA-Seq based profile was developed initially for 5-593 at four development stages to investigate the regulation of pigmentation during seed coat color development. To extend the analysis to other market classes of bean, gene expression data of flavonoid pathway and regulatory genes during the same four stages of seed coat development was captured for the pinto (UI111), pink (Viva), and medium Durango red (UI37) beans using Reverse Transcription quantitative PCR (RT-qPCR) protocols. This study aims to elucidate the molecular mechanisms underlying seed coat formation in common bean by characterizing gene expression profiles associated with flavonoid biosynthesis at different seed coat color developmental stages.

## Materials and methods

Plant materials, flavonoid concentration, RNA extraction, library preparation, and sequencing.

**Fig. 2:**
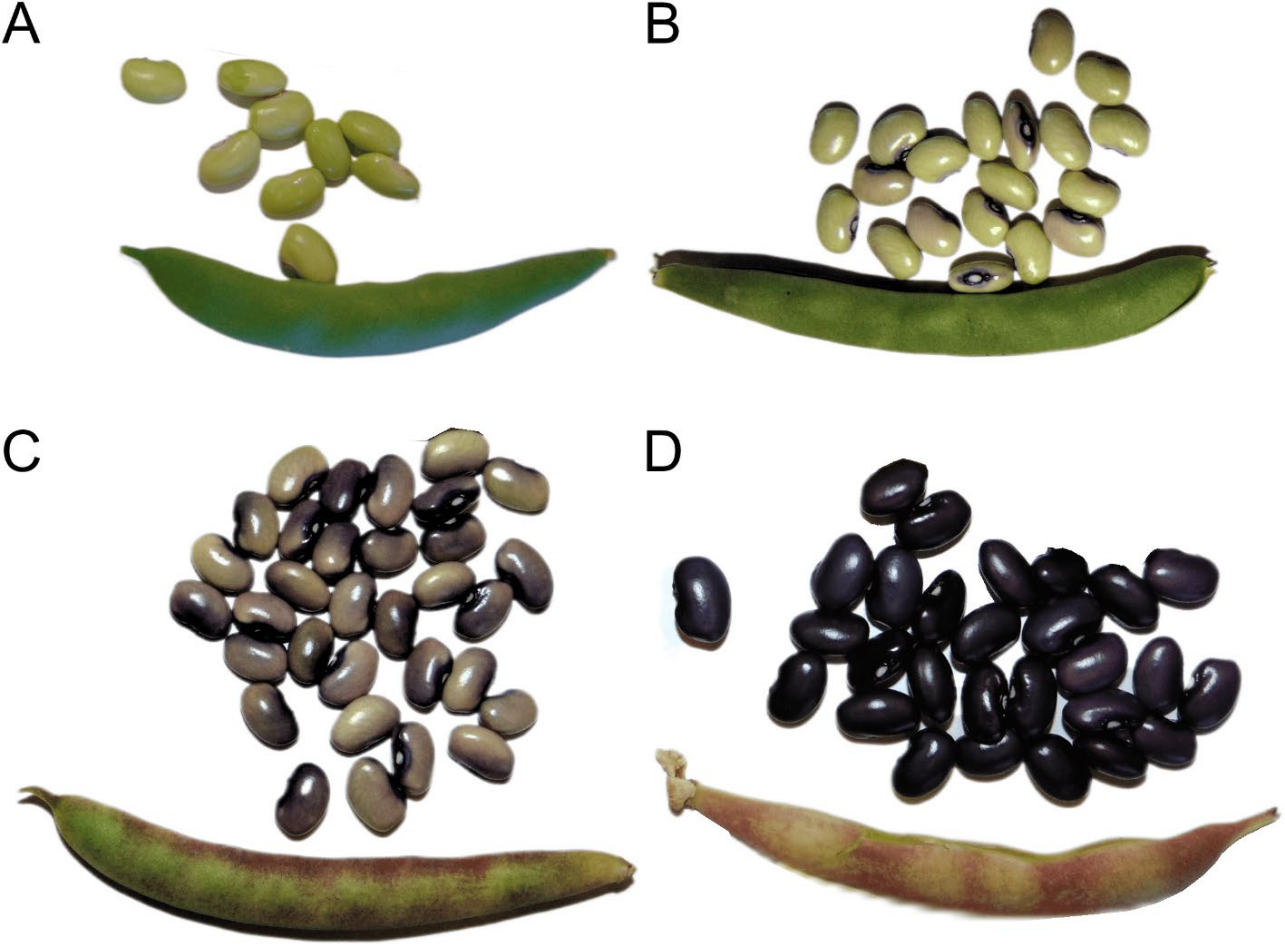
5-593 seed developmental and seed coat pigment expression seeds at the four development stages analyzed using RNA-Seq analysis. **A**) seeds with no pigment (S1), **B**) pigments starting to develop color around the hilum (S2), **C**) about half of black pigments developed on the seed coat (S3), **D**) complete black color developed on the whole seed coat

5-593 (PI 608674) is a black-seeded, purple flower common bean variety developed by Dr. Mark Bassett, University of Florida. 5-593 was used as the recurrent parent to develop a large set of backcross introgression lines in which a single or multiple recessive alleles of seed coat color and pattern genes were introduced into this common genetic background. The concentration of different flavonoids in the seed coat of mature 5-593 seeds was previously published [1] using a LC-MS Quantification protocol. In the present study, immature 5-593 seeds were harvested from greenhouse grown plants. Seed coats, excluding hilum, were extracted from the harvested seeds at four color development stages (Fig. 2A-D) based on seed coat color development. Green seeds with no further color development were considered stage 1 (S1), while stage 2 (S2) comprised seeds in which color development extended beyond the hilum. Purple color formation on half of the seeds were grouped into stage 3 (S3), and full-colored black seeds were considered stage 4 (S4). Three biological replicates of seed coats for each stage were collected and stored separately in tubes, immediately fresh-frozen in liquid nitrogen, and stored at -80⁰ C until RNA extraction.

Total RNA was extracted from seed coats using NucleoSpin^®^ RNA Plant and Fungi Mini-Kit (Macherey-Nagel, Düren, Germany), and quality was assessed using a Qubit fluorometer 4 (Invitrogen INC. Carlsbad, CA). Library preparation and sequencing were performed at the HudsonAlpha Institute for Biotechnology (AL, Huntsville) on an Illumina HiSeq 4000 using the 150 nucleotides (nt) paired-end read configuration to achieve ~ 40 million read pairs per sample. Of the three biological replicates, the first replicates of four stages were sequenced in the first batch, while the other two replicates were sequenced in the second batch.

### Quality control and transcriptome data processing

Low-quality reads (Q ≤ 25), reads shorter than 50 bp, and TruSeq stranded Illumina mRNA-specific adapters were removed using fastp [27]. During reference genome indexing, the genome annotation file was incorporated to provide known exon-exon junctions. High-quality reads were aligned to the *P. vulgaris* L. reference genome 5-593 *v*1.1 (available at https://phytozome-next.jgi.doe.gov/info/Pvulgaris5_593_v1_1) using STAR *v*2.7.8a [28] allowing up to four mismatches across approximately 150 bp sequence and permitting multi-mapping. Read counts were generated with FeatureCounts through Rsubread *v*2.12.3 [29] with gff3 annotations (Pvulgaris5_593_696_v1.1.gene_exons.gff3.gz). FeatureCounts was used to count strand-specific, uniquely aligned reads at the exon level, with read counts subsequently summarized at the gene level. Multi-overlapping features were restricted, ensuring that each read was assigned to only one exon or meta-feature (Table S1).

## Differential gene expression analysis

Differentially expressed genes (DEGs) between the different developmental stages were identified using the DESeq2 package [30] implemented in R *v*4.4.1 [31]. Genes with low expression (read counts < = 10 in at least three samples) were filtered out from the analysis. The model for differential expression included the replicates sequenced at different times in the model matrix (∼batch + condition) to account for batch effects. Batch effects between the sequencing sets were evaluated using principal component analysis (PCA), and the associated batch effect was corrected using the limma package “*removeBatchEffect*” function [32]. The PCA plots were generated to observe clustering across replicates and conditions using batch corrected variance stabilized transformation (VST) value of the normalized counts. Genes with an absolute log_2_-fold value change ≥ 1 and an adjusted *Padj*-value ≤ 0.05 (using the Benjamini and Hochberg correction for multiple testing) were considered significant DEGs. For visualizing and assessing gene expression patterns of genes of interest, transcripts per million (TPM) normalization was used. Heatmaps were generated with the web app flaski [33].

## Functional annotation and pathway enrichment analysis

The primary transcript sequences of 5-593 (*v*1.1) were downloaded from the Phytozome, and functional annotation was performed using eggNOG-mapper *v*2.1.12 [34] (Accessed on April 18, 2024) to identify potential functions based on homology. The resulting GO and KEGG number from eggNOG was used for GO and KEGG enrichment analysis (Table S2). The identified DEGs from each pairwise comparison were subjected to GO enrichment analysis using clusterProfiler *v*4.12.0 [35]. Biological process (BP), molecular function (MF), and cellular component (CC) GO terms with *Padj* < 0.05 were considered significantly enriched for differently expressed genes. DEGs were further subjected to Kyoto Encyclopedia of Gene and Genome (KEGG) database analysis for functional annotation and pathway enrichment analysis via clusterProfiler. A *Padj* < 0.05 value was used to define significantly enriched KEGG pathways.

## Flavonoid pathway and regulatory genes verification in different market classes of beans

Pathway and regulatory genes known to be involved in the flavonoid pathway were selected to investigate their differential expression via RT-qPCR across different dry bean market classes. A total of 14 genes consisting of 12 structural and two regulatory genes were selected. In addition to black-seeded 5-593 genotype, pinto bean “UI111”, medium Durango red bean “UI37”, and pink bean “Viva” were selected for expression analysis (Fig. S1). Three biological replications of seed coats from the four pigment development stages were collected for the RT-qPCR study. Total RNA was isolated from the extracted seed coat tissues, and quality was assessed using a Qubit fluorometer 4. Then 2 µg of purified RNA was reverse transcribed into cDNA using iScript™ Reverse Transcription Supermix (Bio-Rad, US) according to the manufacturer’s instructions. PCR reactions were performed in 15 µl reaction volumes (7.5 µl SsoAdvanced Universal SYBR Green Supermix, 1 µl specific Primer, 2 µl cDNA samples, 3.5 µl RNase-Free H_2_O) according to the manufacturer’s instructions in a CFX Opus 96 Real-time PCR System (Bio-Rad, Inc., Hercules, CA, USA) with the following cycling conditions: 95 °C for 3 min and 45 cycles at 95 °C for 15 s, 55–65 °C for 20 s, and 72 °C for 30 s. Gene-specific primers were designed across two different exons to avoid the possibility of amplifying genomic DNA. Primer specificity was determined by melting curve analysis (Fig. S2). Two technical replicates were performed for each sample. To normalize target gene expression, the histone *H2A9* gene (*HTA9*, Pv5-593.09G200900) was used as an internal control. This gene was selected based on the RNA-Seq data which demonstrated it was constitutively expressed at equal levels across all seed coat stages.

The cycle threshold (Ct) value is defined as the point at which the fluorescence rises above the background fluorescence. The relative expression of mRNA levels of target genes was calculated by the formula 2^(−ΔCt)^, where the ΔCt value of the sample was determined by subtracting the average Ct value of the target gene from the average Ct value of the housekeeping *HTA9* gene. The relative gene expression in fold change (FC) was calculated using the Eq. 2^(−ΔΔCt)^ method [36].

## Results and discussion

### Seed coat transcriptome analyses at different development stages

The mature 5-593 seed coat contains various classes of flavonoids [1]. Among these, delphinidin 3-glucoside is the most predominant flavonoid, followed by the related myricetin derived anthocyanins petunidin 3-glucoside and malvidin 3-glucoside. This result is consistent with the results of Lin et al. and Madrera et al. [15, 16] for other black bean genotypes. Myricetin and myricetin 3-glucoside are the major flavonols, while the flavonols quercetin, quercetin 3-glucoside, kaempferol, and kaempferol 3-O-glucoside are also found in the 5-593 seed coat at lower levels. Traces of catechin and the proanthocyanidin procyanidin B1 were also detected in 5-593 seed coats.

**Fig. 3:**
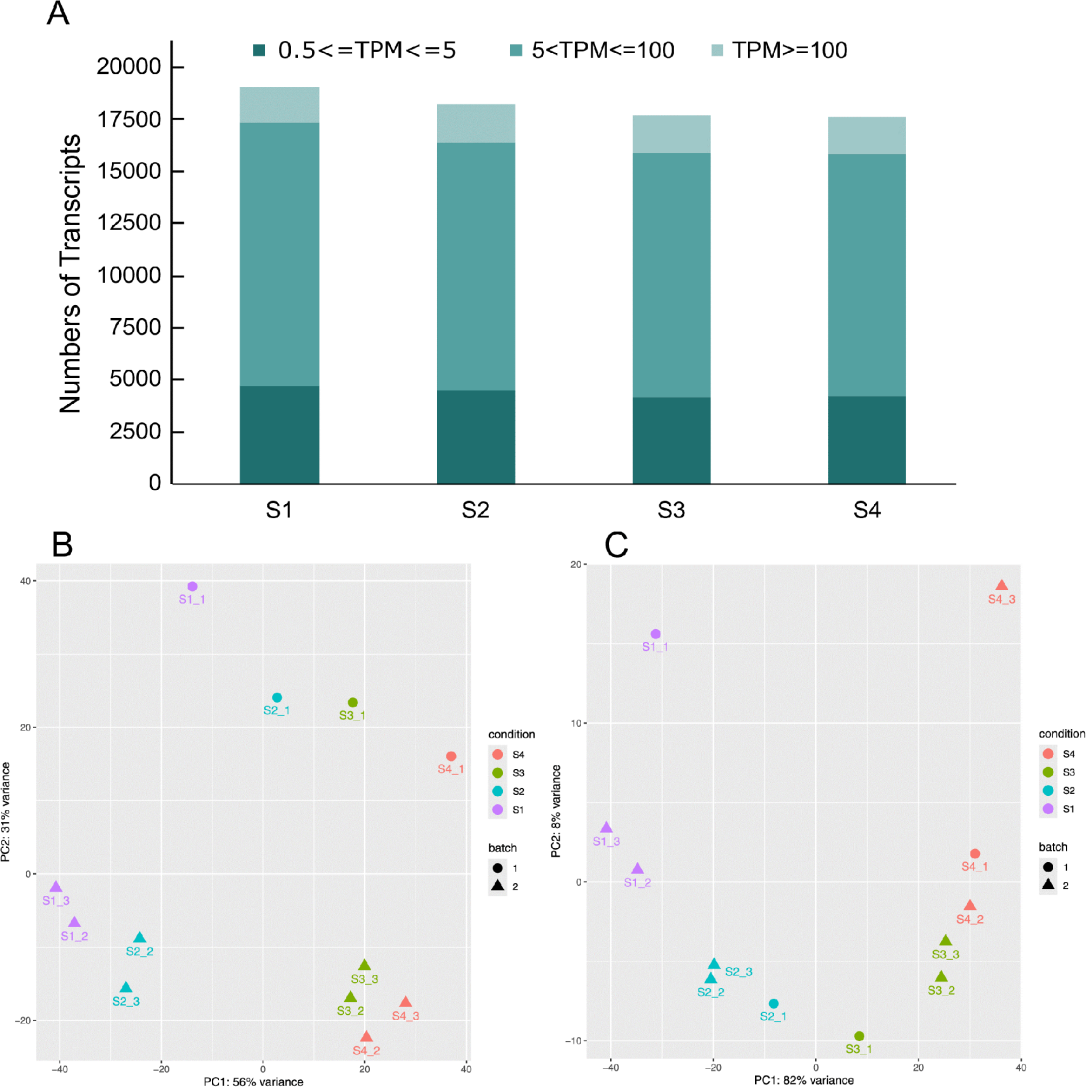
Global gene expression profiling and principal component analysis (PCA) plot to assess the quality of four seed coat pigment development stages of RNA-Seq dataset. **A**) Numbers of detected transcripts in each sample, **B**) PCA plots showed the first two principal component diagram with batch effects, and **C**) removing batch effects from the samples

To explore the molecular basis of the pigment deposition variation in the 5-593 seed coat, RNA-Seq data was collected to generate a global transcriptome profile of genes expressed in the seed coat. The color expression in the seed coat increased over time. Color deposition originated from the hilum and spreads throughout the seed coat as the seed matured until the seed was entirely black (Fig. 2). RNA-Seq analysis was performed on seed coats throughout these four development stages. The raw RNA-Seq reads per library varied from 46.7 to 74.7 million with an average read length of 142 to 145 base pairs (bp) (Table S3). Quality filtering and adapters trimming of raw sequence data resulted in an average GC content of 44% across all samples. The per-base read quality distribution had an average Phred score of 35.8, with the lowest recorded score being 35.4. The numbers of transcripts identified in each sample, expressed as TPM, are shown in Fig. 3A. Genes with normalized reads lower than 0.5 TPM value were removed from the analysis, which resulted in a total of 19,110, 18,240, 17,671, and 17,628 transcripts expressed in S1, S2, S3, and S4, respectively. Among expressed genes across all stages and replicates, approximately 23–25% were in the 0.5–5 TPM range, 66–67% were in the 5–100 TPM range, and 9–10% had greater than 100 TPM value (Fig. 3A). The correlation coefficient of gene expression levels among the biological replicates of four seed coat stages samples was greater than 0.87, indicating the repeatability among biological replicates (Fig. S3).

A Principal Component Analysis (PCA) was constructed to assess the quality and variation among the replicates of the different stages indicated potential batch effects (Fig. 3B). The batch effects were corrected by adding “~batch” in the design function in the DESeq2 package. After batch correction, the PCA plot showed a visible separation between the treatments. The first component accounted for 82%, and the first two components accounted for 90% of the variance and could be used to differentiate the expression of various seed coat stages (Fig. 3C). This indicates a dynamic distribution of gene expression during the transition from a green to black seed coat.

## Identification of differentially expressed genes (DEGs) among different seed coat stages

**Fig. 4:**
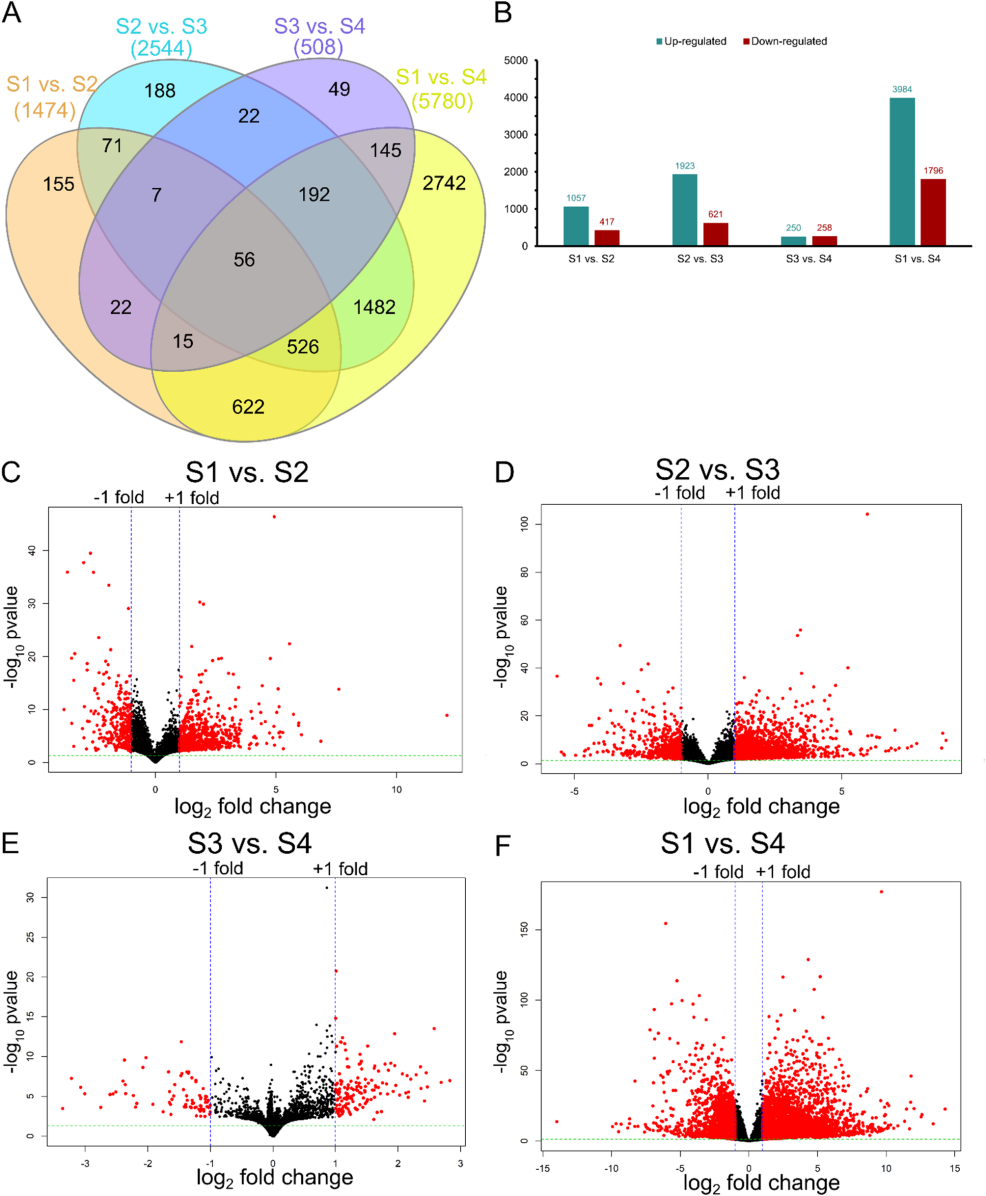
Comparative differential gene expression (DEGs) analysis. **A**) Venn diagrams of DEGs among the four different stages of seed coat; **B**) the number of DEGs up-regulated and down-regulated in four pairwise comparisons. Volcano plot of DEGs between **C**) S1 and S2; **D**) S2 and S3; **E**) S3 and S4; and **F**) S1 and S4 seed coat stages. Each dot represents one gene and red color dots denote significant DEGs that achieved adjusted *P*-value < 0.05

Three seed coat pairwise comparisons, S1 vs. S2, S2 vs. S3, and S3 vs. S4 identified DEGs as the seed coat transitioned from green to black. Additionally, to investigate dynamic gene expression patterns, DEGs were also identified between the earliest S1 and the last S4 seed coat stage (S1 vs. S4). In each pairwise comparison, a total of 1,474 (S1 vs. S2), 2,544 (S2 vs. S3), 508 (S3 vs. S4), and 5,780 (S1 vs. S4) genes were found to be significantly differentially expressed (Fig. 4A-F; Table S4).

Dynamic gene expression was observed across seed coat color development stages, with varying numbers of up-regulated (250 to 3,984) and down-regulated (258 to 1,796) genes identified in each pairwise comparison (Fig. 4B). In general, genes were expressed at higher levels during the early stages of seed coat development. Combining the four pairwise comparisons, 6,294 unique DEGs were detected across different stages of seed coat pigmentation. This finding is consistent with a transcriptome analysis of pigment regulation during testa development in peanut (*Arachis hypogaea*), where 5,452 DEGs across the three developmental stages were identified [37]. Transcriptome analysis of black and white common bean varieties using whole seed, not just seed coat, as study material revealed 10,153 DEGs across three broader seed development stages [26].

### Functional relevance of DEGs

To better classify the biological functions of these DEGs, we implemented GO enrichment analysis to identify GO terms significantly enriched among DEGs in comparison to the genome background. For S1 vs. S2, DEGs were classified into 80 GO categories, including 27 biological processes, 36 cellular components, and 17 molecular functions. DEGs at S2 vs. S3 were divided into 68 GO categories consisting of 25 biological processes (BP), 13 cellular components (CC), and 30 molecular functions (MF); DEGs at S3 vs. S4 were classified only 19 GO categories which include 14 biological processes, one cellular component, and four molecular functions (Fig S4; Table S5).

Among the significantly enriched GO terms, ‘cell division’ (GO:0051301), ‘carbohydrate biosynthetic process’ (GO:0016051), and ‘chloroplast thylakoid’ (GO:0009534) in S1 vs. S2; ‘secondary metabolic process’ (GO:0019748), ‘carbohydrate catabolic process’ (GO:0016052), ‘phenylpropanoid metabolic process’ (GO:0009698), ‘phenylpropanoid biosynthetic process’ (GO:0009699), ‘flavonoid biosynthetic process’ (GO:0009813), and ‘hydrolase activity, acting on glycosyl bonds’ (GO:0016798) in S2 vs. S3; ‘response to water’ (GO:0009415), ‘carbohydrate catabolic process’ (GO:0016052), and ‘abscisic acid-activated signaling pathway’ (GO:0009738) in S3 vs. S4, were mostly predominant. It would be noteworthy to point out that ‘phenylpropanoid metabolic process’ (GO:0009698), ‘phenylpropanoid biosynthetic process’ (GO:0009699), ‘flavonoid metabolic process’ (GO:0009812), and ‘flavonoid biosynthetic process’ (GO:0009813) in the BP category were only significantly enriched when S2 and S3 were compared.

**Fig. 5:**
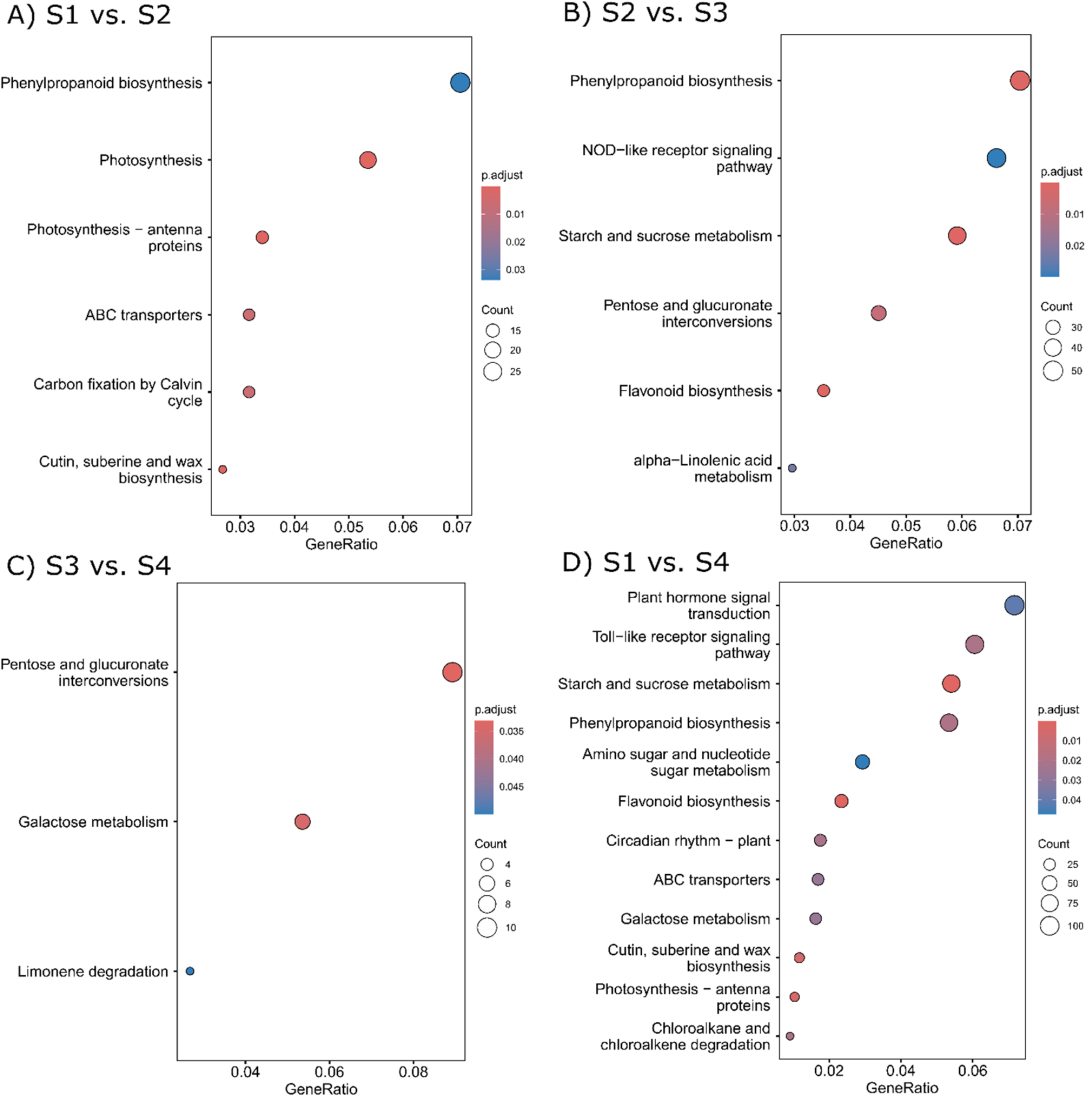
Significantly enriched KEGG pathways (*P* < 0.05) from DEGs in four different seed coat pigment stages. (**A**) S1 vs. S2; (**B**) S2 vs. S3; (**C**) S3 vs. S4; and (**D**) S1 vs. S4

The KEGG analysis of the DEGs obtained from different pairwise comparisons revealed that 20 unique KEGG pathways were significantly enriched (Fig. 5; Table S6). Among the significantly enriched pathways, 102 (S1 vs. S2), 217 (S2 vs. S3), and 19 (S3 vs. S4) DEGs were assigned to these alignments with a total of six, six, and three, respectively, enriched KEGG pathways. The most predominant and prevalent enriched KEGG pathways were associated with sucrose metabolism (ko00500), phenylpropanoid biosynthesis (ko00940), flavonoid biosynthesis (ko00941), plant hormone signal transduction (ko04075), galactose metabolism (ko00052), and NOD-like receptor signaling pathway (ko04621. To understand the biosynthesis and metabolism of bean seed coat pigmentation, DEGs enriched in the phenylpropanoid biosynthesis (ko00940) and flavonoid biosynthesis (ko00941), were studied further. Phenylpropanoid biosynthesis pathways and flavonoid biosynthesis pathways were significantly enriched in S1 vs. S2 and S2 vs. S3, while flavonoid biosynthesis pathways were also enriched in the S2 vs. S3 comparisons. Functional annotation and KEGG analysis revealed a total of 29 genes were involved in phenylpropanoid biosynthesis were differentially expressed between seed coat color development stages S1 and S2; while 50 genes (31 new) involved in phenylpropanoid biosynthesis and 25 in flavonoid biosynthesis were differentially expressed in S2 compared to S3 stage (Table S6). Among these genes, 10 and 31 DEGs belong to phenylpropanoid biosynthesis were uniquely categorized and expressed in S1 vs. S2 and S2 vs. S3 comparisons, respectively, indicating their dynamic expression pattern over the course of seed coat color development. Flavonoid biosynthesis (ko00941) pathway comprising 25 DEGs only enriched in S2 vs. S3 stage as color deposition in the seed coat started around stage S2. Therefore, genes involved in the phenylpropanoid biosynthesis pathway turned on at the earlier stages of seed coat to convert primary phenylalanine substrate to hydroxycinnamic acid. The next several steps to synthesize different classes of flavonoids are regulated by various flavonoid pathways and regulatory genes that are enriched in S2 vs. S3 stage, followed by their constitutive expression from S3 to S4 seed coat stage.

### Verification of DEGs during seed coat color development

**Fig. 6:**
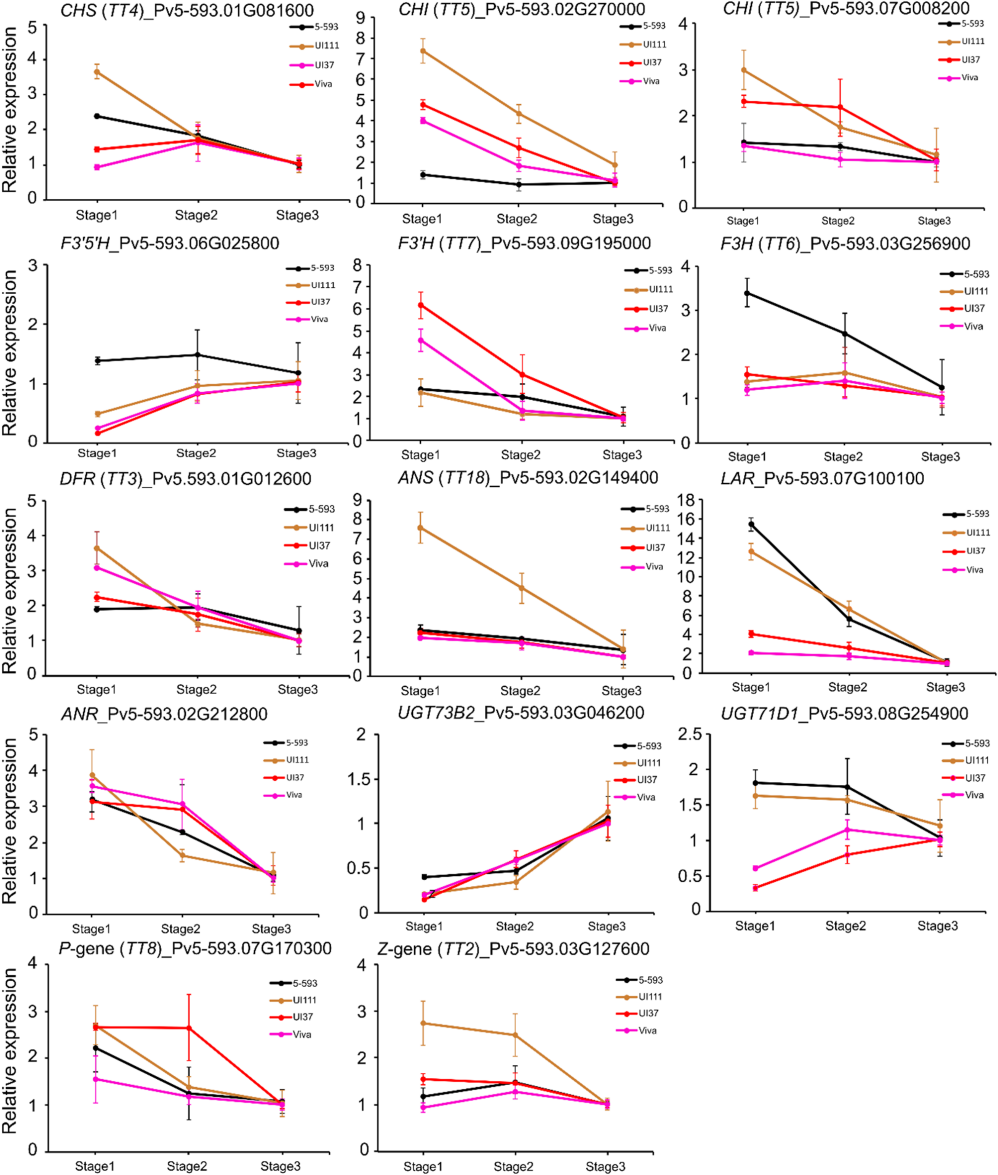
RT-qPCR verification and analysis of 12 structural and two regulatory genes involved in the flavonoid biosynthesis pathway in four different bean market classes including 5-593 for stages S1, S2, and S3 of seed coat development. The x-axis shows the three different seed coat stages, while y-axis represents the relative gene expression levels shown as ΔΔCt values relative to S3

To verify the gene expression profiles identified by RNA-Seq data,14 genes with TPM values ≥ 5 were selected for RT-qPCR analysis (Fig. 6, Fig. S5) using gene-specific primers (Table S7). Since the amount of RNA extracted from S4 seed coats was below the concentration needed for accurate RT-qPCR analysis, that stage was excluded from the RT-qPCR analysis. The fold change in expression levels of these genes at the S1 and S2 stages was analyzed with reference to the S3 seed coat stages.

### Analysis of DEGs involved in seed coat color formation in different bean market classes

**Fig. 7:**
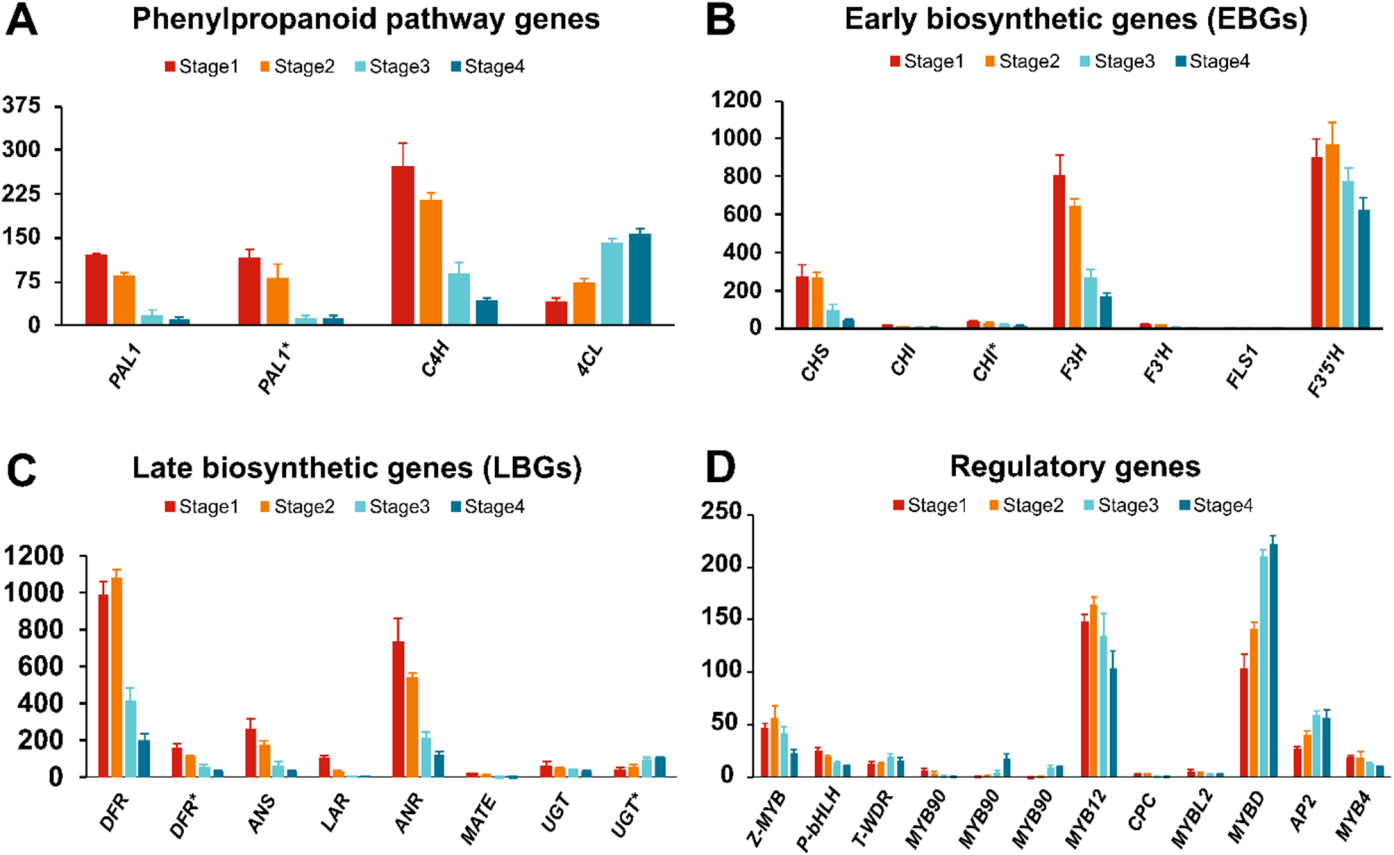
The expression profiles of the key phenylpropanoid pathway (**A**), early biosynthetic (**B**), late biosynthetic (**C**), and regulatory (**D**) genes involved in the flavonoid biosynthesis pathway during four seed coat pigment development stages in 5-593. The mean RNA-Seq TPM value of three biological replicates in each stages sample is found on the Y-axis. Error bar indicates standard error for reach stage. The designated gene symbols correspond to 5-593 gene models, and their expression patterns were presented in Table S10

Since the primary objective was to investigate the underlying molecular mechanisms in common bean seed coat pigmentation, the phenylpropanoid biosynthesis and flavonoid biosynthesis pathway genes were analyzed in detail. Differentially expressed genes encoding enzymes involved in the phenylpropanoid biosynthesis, phenylalanine metabolism, flavonoid biosynthesis, and plant hormone signal transduction pathways were enriched during seed coat pigment development. A total of 112 DEGs, corresponding to 87 and 36 belonging to phenylpropanoid biosynthesis and flavonoid biosynthesis pathways, respectively, were identified to be involved in seed coat color pigmentation in common bean (Table S8). Among these, 29 DEGs were known to be involved in seed coat pigmentation and are considered as essential candidate genes associated with bean seed coat color development (Table 1; Fig. 7A-C).

**Table 1:** List of the important candidate genes differentially expressed in various 5-593 seed coat pigment development stages enriched in the phenylpropanoid and flavonoid biosynthesis pathways

KEGG Pathways	5-593 gene model	Gene Name	Functional annotation
Flavonoid biosynthesis	Pv5-593.01G012600, Pv5-593.01G012700	*DFR*,* M318*,* TT3*	dihydroflavonol 4-reductase
Phenylpropanoid biosynthesis	Pv5-593.01G176600, Pv5-593.01G176700, Pv5-593.07G149800	*PAL1*	Phenylalanine ammonia-lyase
Flavonoid biosynthesis	Pv5-593.01G069300, Pv5-593.01G081600, Pv5-593.02G038700, Pv5-593.02G038800, Pv5-593.02G038900, Pv5-593.02G039000, Pv5-593.02G039100, Pv5-593.02G039200, Pv5-593.02G039300, Pv5-593.02G039400, Pv5-593.02G039500, Pv5-593.02G181100	*CHS*,* TT4*	Naringenin-chalcone synthase / Flavonone synthase
Phenylpropanoid biosynthesis and Flavonoid biosynthesis	Pv5-593.06G086700, Pv5-593.08G239900	*ATC4H*,* C4H*,* CYP73A5*,* REF3*	Trans-cinnamate 4-monooxygenase / Cinnamic acid 4-monooxygenase
Flavonoid biosynthesis	Pv5-593.03G256900	*F3\‘H*,* F3H*,* TT6*	naringenin 3-dioxygenase
Flavonoid biosynthesis	Pv5-593.02G149400	*ANS*,* LDOX*,* TDS4*,* TT18*	leucoanthocyanidin dioxygenase
Flavonoid biosynthesis	Pv5-593.02G212800	*BAN*	anthocyanidin reductase
Flavonoid biosynthesis	Pv5-593.02G270000, Pv5-593.07G008200, Pv5-593.07G008300	*A11*,* CFI*,* CHI*,* TT5*	chalcone isomerase
Flavonoid biosynthesis	Pv5-593.09G195000	*CYP75B1*,* F3’H*,* D501*,* TT7*	flavonoid 3’-monooxygenase
Flavonoid biosynthesis	Pv5-593.08G160900	*ATFLS1*,* FLS*,* FLS1*	flavonol synthase
Phenylpropanoid biosynthesis	Pv5-593.11G084700	*4CL*	4-coumarate–CoA ligase
Flavonoid biosynthesis	Pv5-593.07G100100	*LAR*	Leucocyanidin reductase

Seed coat color in *P. vulgaris* is primarily determined by flavonoids including the flavones, flavonols, anthocyanins, and proanthocyanidins that color leaves, flowers, and seeds [38–40]. All flavonoid classes are synthesized from phenylalanine through the phenylpropanoid pathway by three enzymatic reactions catalyzed by phenylalanine ammonia lyase (*PAL*), cinnamic acid 4-hydroxylase (*C4H*), and 4-coumarate-CoA ligase (*4CL*) [41] (Fig. 1). Here we showed that in 5-593 three *PAL1* genes (Pv5-593.01G176600, Pv5-593.01G176700, Pv5-593.07G149800), *4CL* (Pv5-593.11G084700), and *C4H* (Pv5-593.06G086700, Pv5-593.08G239900)] were differentially enriched. Among the three 5-593 *PAL1* genes, Pv5-593.07G149800 had the lowest expression, and the level of expression decreased from stage S1 to stage S4. Both *C4H* genes were down-regulated, and *4CL* gene was up-regulated as the seed coat pigmentation advanced. Among the two *C4H* genes, gene model Pv5-593.08G239900 showed a higher expression level than Pv5-593.06G08670.

The flavonoid branch of the phenylpropanoid pathway leads to flavonoid production (Fig. 1). Through KEGG enrichment analysis and functional annotation, 36 DEGs of the anthocyanin/flavonoid biosynthesis pathway were identified. Among these, several known flavonoid pathway genes including *CHS*, *CFI*, *CHI*, *F3H*, *F3*′*H*, *F3*′*5*′*H*, *FLS1*, *DFR*, *ANS*, *LAR*, *ANR*, *UGT* were identified as DEGs. These findings correlate with the seed color RNA-Seq analysis results in black seeded mung bean (*Vigna radiata*) [42], peanut (*Arachis hypogaea*) [37], and *Brassica rapa* [43]. The 36 flavonoid biosynthesis pathway DEGs included two dihydroflavonol 4-reductase (*DFR*) genes, 12 naringenin-chalcone synthase (*CHS*) genes, one naringenin 3-dioxygenase (*F3H*), three chalcone isomerase (*CHI*) genes, and one flavonol synthase 1 (*FLS1*), flavonoid 3’-monooxygenase, leucoanthocyanidin dioxygenase (*ANS*), anthocyanidin reductase (*ANR*), and leucocyanidin reductase (*LAR*) encoding genes. Several DEGs encoding three trans-cinnamate 4-monooxygenase, and two caffeoyl-CoA O-methyltransferase were found to be included in both phenylpropanoid and flavonoid biosynthesis pathways.

For 5-593, the expression level of twelve flavonoid pathway and two pathway regulatory genes, measured by RT-qPCR, and expressed as fold change with reference to S3 stage, were consistent with the results obtained by RNA-Seq data (expressed as mean TPM values of three biological replicates). RT-qPCR analyses of the same 14 genes were also performed on the first three seed coat development stages of genotypes representing the pinto (UI111), pink (Viva), and medium Durango red (UI37) market classes (Fig. 6). For 5-593, the RNA-Seq TPM value of the *CHS* gene Pv5-593.01G081600 was highest at the S1 stage (TPM = 278.8), and then gradually decreased to 98.9 at S3. This expression pattern was similar to the RT-qPCR expression data. This expression pattern was also noted for UI111. However, UI37 and Viva showed higher expression in S2 than in the S1 and S3 seed coat stages. For UI111, UI37, and Viva, the expression level of *DFR* gene model (Pv5-593.01G012600) was higher at the earlier stage, followed by lower expression at later development stages while its expression peaked at S2 for 5-593. For all genotypes, the expression pattern of the two *CHI* genes and the *F3H*, *DFR*, *ANS*, *ANR*, and *F3’H* gene models were characterized by a higher expression at the early S1 and S2 stages before decreasing at the later S3 stage. The results also revealed that the two UDP-glucosyl transferase genes (Pv5-593.03G046200, Pv5-593.08G254900) exhibited opposite expression patterns during the seed coat pigment development process. For example, Pv5-593.03G046200 gene expression decreased from S1 to S2-S3 stages for all the four bean market classes. In contrast, the Pv5-593.08G254900 level increased from the early to later stage for 5-593 and UI111 genotypes, which is quite the opposite for the medium red UI37 and pink Viva genotypes. Two regulatory genes, *P* (Pv5-593.07G170300) [19] and *Z* (Pv5-593.03G127600) [24] were characterized by higher expression levels at the early stage, followed by reduced expression at the later stages of seed coat pigmentation (Fig. 6).

Black bean seed coats primarily contain the anthocyanin delphinidin 3-O-glucoside (D3G) and its O-methylated derivates malvidin 3-glucoside, and petunidin 3-glucoside. A functioning flavonoid 3’,5’-hydroxylase (*F3’5’H*) enzyme, encoded by the classic *V* gene (Pv5-593.06G025800) [1], is the first committed enzyme in the flavonoid pathway which leads towards the biosynthesis of delphinidin 3-O-glucoside and its derivatives (Fig. 1). This gene was strongly expressed in 5-593 at the early stage (S1: TPM 905.29 and S2: TPM 975.98) and decreased gradually over time (S3: TPM 777.42; S4: TPM 622.56), suggesting a moderate down-regulation of this gene as the seed coat pigment development progress. 5-593 carries the dominant *V* gene. UI111, UI37, and Viva carry the recessive *v* allele and do not produce delphinidin anthocyanins since it requires a functional F3′5′H protein [9, 12]. Recently, it was shown that the recessive *v* allele lacks multiple functional domains necessary for the *F3’5’H* protein to be active in the pathway [1]. The three recessive genotypes showed a significantly lower level of expression at the early stages compared to 5-593, but by stage S3 the gene was transcribed to a similar level as 5-593 (Fig. 6). The fact that the gene is expressed in all four genotypes demonstrates that recessive allele is transcribed, but the protein is non-functional. This observation supports the conclusion that mutations in the coding, and not the regulatory region, of the gene are responsible for the lack of dihydromyricetin-derived anthocyanins in the pinto, pink, and medium-sized Durango red market classes.

*DFR* converts dihydroflavonols to leucoanthocyanidins which are precursors to anthocyanins, flavan-3-ols, and proanthocyanidins (PAs) [44]. Three copies of *DFR* genes (Pv5-593.01G012600, Pv5-593.01G012700, Pv5-593.07G243800) are found in the common bean genome. The *DFR* gene model Pv5-593.01G012600 had a higher expression in the seed coat of 5-593 at earlier stages than Pv5-593.01G012700, whereas Pv5-593.07G243800 had no expression at any of the stages. The expression of Pv5-593.01G012600 was highest at early stages for the UI111, Viva, and UI37 and its expression in 5-593 was lower. This DFR copy was expressed at equal levels for all four genotypes at stages S2 and S3. In 5-593, the only *ANS* gene (Pv5-593.02G149400) was expressed 1 to 3 times higher at early stages compared to later seed coat stages, consistent with the findings reported by Tapia et al. [26]. This gene is expressed at much lower levels in the other market classes. Two UGT encoding genes were significantly induced among different stages. Among these, Pv5-593.03G046200 showed a decreased expression level and Pv5-593.08G254900 showed increased expression at the later stages of seed coat development. *LAR* is the first committed enzyme for the production of proanthocyanidins, and converts leucoanthocyanidins to catechin, afzelechin, or gallocatechin, depending on the substrate. *LAR* expression for 5-593 and UI111 was six and four times higher than Viva and UI37 at stage S1, and their expression level dropped to the same level as these two genotypes by stage S3 (Fig. 6). The expression levels of anthocyanidin reductase (*ANR*) for the four genotypes were high at the early stage of seed coat development and dropped significantly for all genotypes by stage S3.

### Regulatory genes involved in the flavonoid gene expression

The genes encoding the enzymes of the flavonoid pathway are regulated by a wide array of proteins representing the MYB, bHLH, WDR (WD40 repeat), bZIP, NAC, MADS-box, and GRF-like transcription factors families. The primary focus here is on the MYB, bHLH, and WDR families with a special reference to those genes identified in Arabidopsis as a reference point. In pairwise comparison across different seed coat stages, 56 MYBs, 31 bHLHs, and nine WD40s were differentially expressed (Fig. S6; Table S9). The results showed that two bHLH genes were up-regulated (2/3) in S1 vs. S2, 18 were up-regulated (18/28), all eight bHLH genes were up-regulated in S2 vs. S3, and two bHLH down-regulated in S3 vs. S4 (2/2) comparison. Among the 56 MYBs DEGs, the majority were down-regulated (41/56) as the seed coat stage advances. The results also revealed that DEGs belonging to the WD40s family were down-regulated (9/9) during the seed coat pigmentation process. In Arabidopsis, *MYB12* is a key regulator of the expression of EBGs of the pathway are controlled redundantly by several MYB homologous genes including *MYB12* [45]. Pv5-593.06G121300 (*PvMYB12*), the sole *MYB12* homolog in bean (blastp value = 1.00e-72; 75% identity), is stably expressed across all seed coat stages with a relatively high expression level (Fig. 7D; Table S10). In contrast, the LBGs of the flavonoid pathway are controlled by the MBW ternary complex consisting of MYB, bHLH, and WDR protein [44]. This complex acts as a positive or negative regulator depending upon the specific MYB proteins that either are part of the complex [46] or interact in a way that disrupts the complex. Recent gene cloning and computational modeling identified three common bean gene models (Pv5-593.03G127600, Pv5-593.07G170300 and Pv5-593.09G04730) encoding proteins homologous to the Arabidopsis MBW proteins (*TT2*, *TT8*, and *TTG1*, respectively), which regulates the expression of the LBGs anthocyanin genes associated with anthocyanin biosynthesis [19, 24]. These three genes encode *Z*, whose recessive allele is necessary for partial seed coat patterning, *P*, whose dominant allele is required for any seed coat color expression, and *T*, whose recessive allele is required for any partial seed coat pattern. The expression level for Pv5-593.03G127600 (*Z-MYB*) and Pv5-593.07G170300 (*P-bHLH*) peaked early in seed coat development and showed significant decreases in the late two stages. By contrast, the Pv5-593.09G04730 (*T-WDR*) expression was low in the first two stages and was highest during later stages (Fig. 7D).

While the MBW complex is a positive regulator of flavonoid expression, negative regulators have been identified, and homologs of these express similar regulatory effects in multiple species [47]. The R3 MYB protein *MYBL2* is a major negative regulator that interacts with the MBW complex resulting in significant decreased expression of the LBGs *DFR* and *ANS* and reduced proanthocyanidin biosynthesis [48]. The 5-593 homolog to *MYBL2* (Pv5-593.02G090900, *PvMYBL2*; blastp value = 9.13e-36; 69% identity) contains the critical terminal repressive motif TLLLFQ and is expressed at modest levels that are maintained during all stages of seed coat development. Arabidopsis *MYBD* (AT1G70000) is a positive regulator of anthocyanin synthesis. It counteracts the *MYBL2* repression by binding to the *MYBL2* promoter and inhibiting the expression of the gene [49] which in turn maintains the expression of LBGs. Pv5-593.02G078700 (*PvMYBD*) is a homolog (blastp value = 4.02e-73; 73% identity) of Arabidopsis *MYBD*. *PvMYBD* was up-regulated from stages S1 to S4 and may keep the *PvMYBL2* level low so that dihydromyricetin can be produced by *DFR* and act as substrate for eventual accumulation of the anthocyanin delphinidin 3-O-glucoside, the most abundant flavonoid in 5-593. More recently, Arabidopsis *AP2*, a regulator of seed coat development [50], was also identified as a major negative regulator of proanthocyanin accumulation by promoting the expression of the negative regulator *MYBL2*. *AP2* and *MYBL2* interact with the bHLH component of the MBW complex and disrupt its function as a LBG activator. Pv5-593.02G016100 (*PvAP2*), a strong *AP2* homolog (blastP value = 9.2E-118; 70% identity) was expressed at relatively high levels in S1 and was significantly increased in S2 at expression levels that were maintained during the remainder of seed coat development (Fig. 7D; Table S10). The expression patterns of the negative regulators *PvAP2* and *PvMYBL2* may account for low levels of proanthocyanidins found in 5-593 seed coats.

### Transcriptional regulation of hormone signal transduction pathway genes during bean seed coat development

**Fig. 8:**
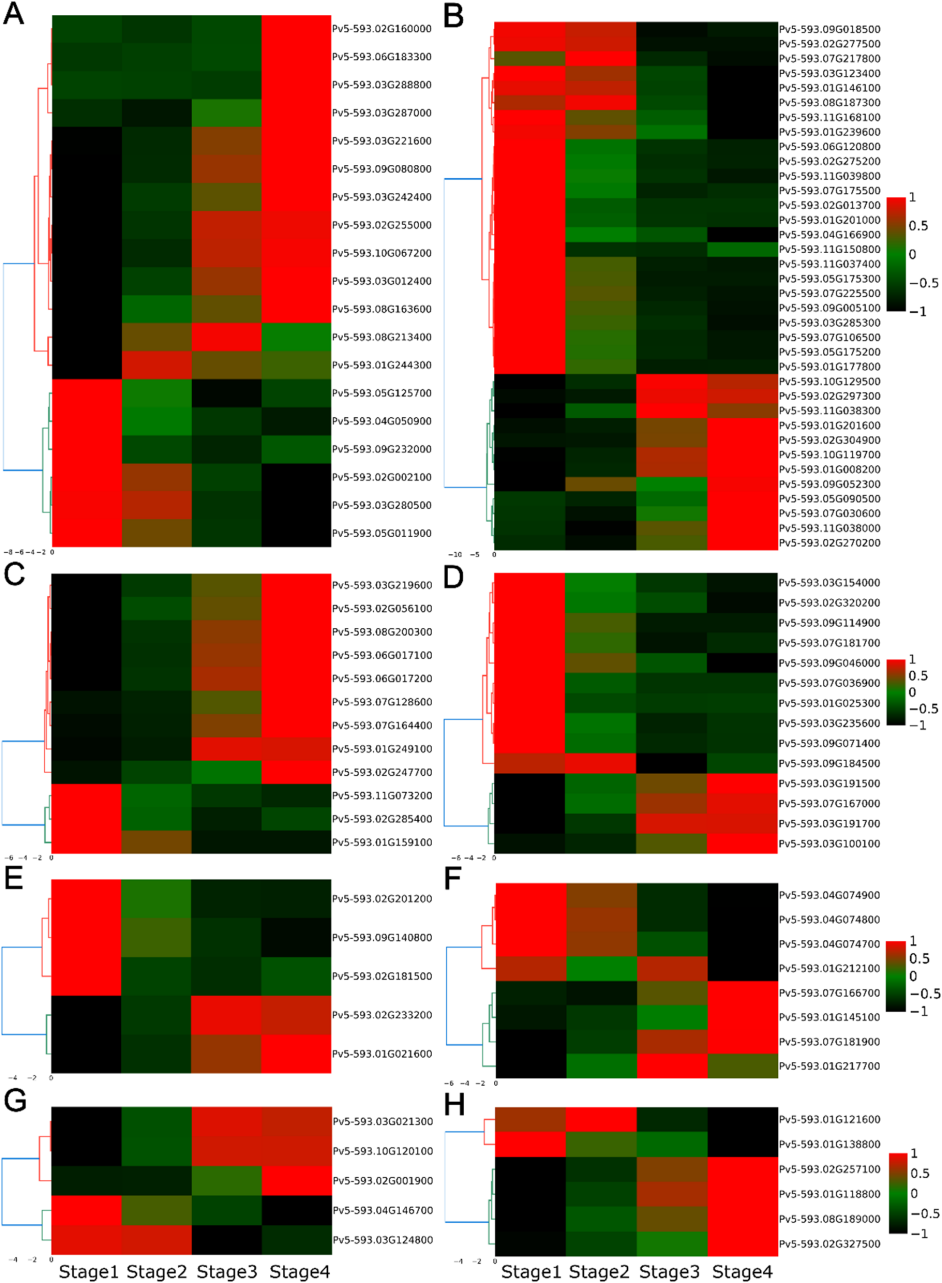
The expression profiles of plant hormone signal transduction DEGs during seed coat pigment development in common bean. **A**) 19 ABA pathway genes; **B**) 36 AUX pathway genes; (**C**) 12 ethylene (ET) signal transduction pathway genes; (**D**) 14 cytokinin (CTK) pathway genes; (**E**) Five brassinosteroid (BR) pathway genes; (**F**) Eight gibberellin (GA) signal transduction pathway genes; (**G**) Five jasmonic acid (JA) signal transduction pathway genes; (**H**) Six salicylic acid (SA) signal transduction pathway genes. Gene expression was scaled in this analysis using TPM Z-scores based on the mean value of three biological replicates in the heatmap. The key is located on the right-hand side in each case with TPM values increasing from black, green to red

Plant hormones can positively or negatively regulate anthocyanin biosynthesis during seed coat development and pigmentation [40, 51–53]. Differential expression and functional enrichment analysis revealed 111 DEGs assigned to the plant hormone signaling pathway enriched in the S1 vs. S4 seed coat stages comparison (Fig. 8; Table S11). Among these, 36 DEGs were associated with the auxin (AUX) signaling pathway, while 19, 14, and 12 DEGs were involved in the abscisic acid (ABA), cytokinin (CTK), and ethylene (ET) signaling pathways; 11 each were implicated with the gibberellin (GA) and jasmonic acid (JA) signaling pathways, and six and five DEGs were found in salicylic (SA) and brassinosteroid (BR) signaling transduction pathway, respectively. Twenty-four (24) AUX pathway DEGs were down-regulated (24/36), which is similar to the decreased expression levels of the majority of CTK signal pathway DEGs (10 out of 14) and BR pathway DEGs (3/5) over the course of seed coat color development. On the contrary, expression data of other hormone signal transduction pathways, such as 13 (13/19) of the DEGs involved in the ABA signaling pathway, nine DEGs (9/12) in the ET pathway, and seven (7/11) of each in the GA and JA signaling pathway, respectively, were up-regulated throughout the growth and seed coat pigmentation development advances (Fig. 8; Table S11). Based on the observed results, we suggest that the AUX, CTK, and BR signaling pathways acted antagonistically to influence common bean seed coat color development with respect to the stress hormone pathway, which aligns with the transcriptome analyses in peanut testa pigmentation findings [37].

In this study, a comprehensive transcriptome analyses were employed to investigate the regulatory mechanisms underlying seed coat pigmentation in common bean. Utilizing four distinct developmental stages of seed coat tissues, we identified 6,294 differentially expressed genes (DEGs) through pairwise comparisons. As anticipated, KEGG pathway enrichment analysis highlighted the phenylpropanoid biosynthesis (ko00940) and flavonoid biosynthesis (ko00941) pathways as key biological processes involved in determining seed coat color in common bean. Genes involved in the phenylpropanoid biosynthesis pathway upregulated in the early stages of seed coat development convert primary phenylalanine substrate into hydroxycinnamic acids. Subsequent steps involved in the synthesis of different classes of flavonoids are regulated by enzymes encoded by genes such as chalcone synthase (*CHS*), chalcone isomerase (*CHI*), flavanone 3-hydroxylase (*F3H*), flavonoid 3’,5’-hydroxylase (*F3’5’H*), flavonoid 3’-hydroxylase 1 (*F3’H*), flavanone synthase (*FLS1*), dihydroflavonol 4-reductase (*DFR*), anthocyanidin synthase (*ANS*), leucoanthocyanidin reductase (*LAR*), anthocyanidin reductase (*ANR*), and UDP-glucose: flavonoid 3-O-glucosyltransferase (*UGT*), were differentially regulated between stages S2 and S3, followed by constitutive expression from stage S3 to S4. Twenty-nine (29) DEGs encoding these key enzymes were identified as candidate genes involved in increasing pigment accumulation. Furthermore, four MYB transcription factors were identified as potential regulators of seed coat pigmentation. In conclusion, this comprehensive transcriptome analysis provides a valuable genomic resource for the bean research community and offers molecular insights into the transcriptomic network underpinning seed coat color development in common bean.

## Electronic supplementary material

Below is the link to the electronic supplementary material.

Supplementary Material 1: Seed developmental stages and seed coat pigment acquisition of collected samples at three different stages (S1, S2, S3) for RT-qPCR analysis. A) samples of UI111 genotype consisted of green seeds with no stripe (S1), green seeds with stripe (S2), yellow seeds with stripe (S3); B) UI37 genotype comprised of green immature seeds (S1), red color started to develop on the seed coat (S2), and red color retained completely on the seed coat (S3); C) Viva genotypes with no color on seeds, mostly white (S1), pink color started to develop on the seed coat (S2), seeds retained complete pink color (S3)

Supplementary Material 2: Melt curve analysis to determine the gene specific primers to be used in RT-qPCR analysis. Single peak in the melt curve plot indicates the deigned primer is gene specific

Supplementary Material 3: Correlation heatmap between the biological replicates of different seed coat stages tissues developed based on the raw reads from RNA-Seq data. S1, S2, S3, and S4 represent the RNA sample collected from seed coat stage 1, 2, 3, and 4 respectively and the numeric value after “_” sign refers to the assigned biological replicate. Blue and red colors indicate positive correlation and negative correlation, respectively

Supplementary Material 4: Gene Ontology (GO) annotation and over-representation analysis of differentially expressed genes in five pairwise comparisons. Dot plots represent GO annotation of DEGs from A) S1 vs. S2, B) S2 vs. S3, C) S3 vs. S4, and D) S1 vs. S4 comparisons into three categories: biological processes (BP), cellular components (CC), and molecular functions (MF)

Supplementary Material 5: Validation of 5-593 RNA-Seq data by RT-qPCR through the comparison of the gene expression ratios from qRT-PCR and RNA-Seq data. The correlation between RNA-Seq and RT-qPCR of the fold change in expression levels of the 14 genes in 5-593

Supplementary Material 6: The expression profiles of MYB-bHLH-WD40 DEGs during seed coat pigment development. (A) 56 MYB family genes; (B) 31 bHLH family genes; and (C) 9 WD40 family genes. Gene expression was scaled in this analysis using TPM Z-scores based on the mean value of three biological replicates in the heatmap. The key is located on the right-hand side in each case with TPM values increasing from black, green to red

Supplementary Material 7: Scripts and pseudocode for RNA-Seq data preprocessing, aligning, and counting reads

Supplementary Material 8: List of 5-593 gene models with the identified GO and KEGG pathway identifiers obtained from eggNOG annotation

Supplementary Material 9: Global statistics of RNA-seq data and mapping results in four different seed coat pigment acquisition samples in 5-593

Supplementary Material 10: List of differentially expressed genes (DEGs) identified in all pairwise comparisons among different seed coat stages samples in 5-593

Supplementary Material 11: List of significantly enriched GO processes of DEGs during seed coat pigmentation of common bean development

Supplementary Material 12: List of significantly enriched KEGG pathways of DEGs during seed coat pigmentation of common bean development

Supplementary Material 13: Sequences of the primers used in the study

Supplementary Material 14: List of DEGs significantly enriched in the phenylpropanoid biosynthesis and flavonoid biosynthesis across four different seed coat pigment stages in 5-593

Supplementary Material 15: List of differentially identified bHLH, MYB, and WD40 transcription factor genes and it's TPM value

Supplementary Material 16: List of pathway and regulatory genes involved in regulating flavonoid biosynthesis and expression pattern in the 5-593 genome

Supplementary Material 17: List of differentially expressed genes, annotations, and expressions enriched in plant hormone signal transduction pathway in different seed coat stages in 5-593

## Data Availability

All the data generated or analyzed in this research are included in this published article (and its supplementary information files). The raw RNA-Seq data are available in the NCBI repository by the BioProject ID: PRJNA1200069.

## Abbreviations

DEG: Differentially expressed gene
KEGG: Kyoto Encyclopedia of Genes and Genomes
EBG: Early biosynthetic gene
CHS: Naringenin-chalcone synthase
CHI: Chalcone isomerase
F3H: Naringenin 3-dioxygenase
FLS: Flavonol synthase 1
LBG: Late biosynthetic gene
DFR: Dihydroflavonol 4-reductase
ANS: Anthocyanidin synthase/Leucoanthocyanidin dioxygenase
LAR: Leucoanthocyanidin reductase
ANR: Anthocyanidin reductase
UGT: UDP-glucose: flavonoid 3-O-glucosyltransferase
F3′5′H: Flavonoid 3′5′ hydroxylase
PCA: Principal component analysis
VST: Variance stabilized transformation
GO: Gene ontology
BP: Biological process
CC: Cellular component
MF: Molecular functions
TPM: Transcripts per million
PAL: Phenylalanine ammonia lyase
C4H: Cinnamic acid 4-hydroxylase
4CL: 4-coumarate-CoA ligase
Pas: Proanthocyanidins
AUX: Auxin
ABA: Abscisic acid
CTK: Cytokinin
ET: Ethylene
GA: Gibberellin acid
JA: Jasmonic acid
SA: Salicylic acid
BR: Brassinosteroid
