# Supplementary figures and images for: Seed coat transcriptomic profiling of 5-593, a genotype important for genetic studies of seed coat color and patterning in common bean (Phaseolus vulgaris L.)
Source: BMC Plant Biol. 2025 Mar 5;25:284. doi: 10.1186/s12870-025-06282-7 (PMC11881399; doi:10.1186/s12870-025-06282-7)

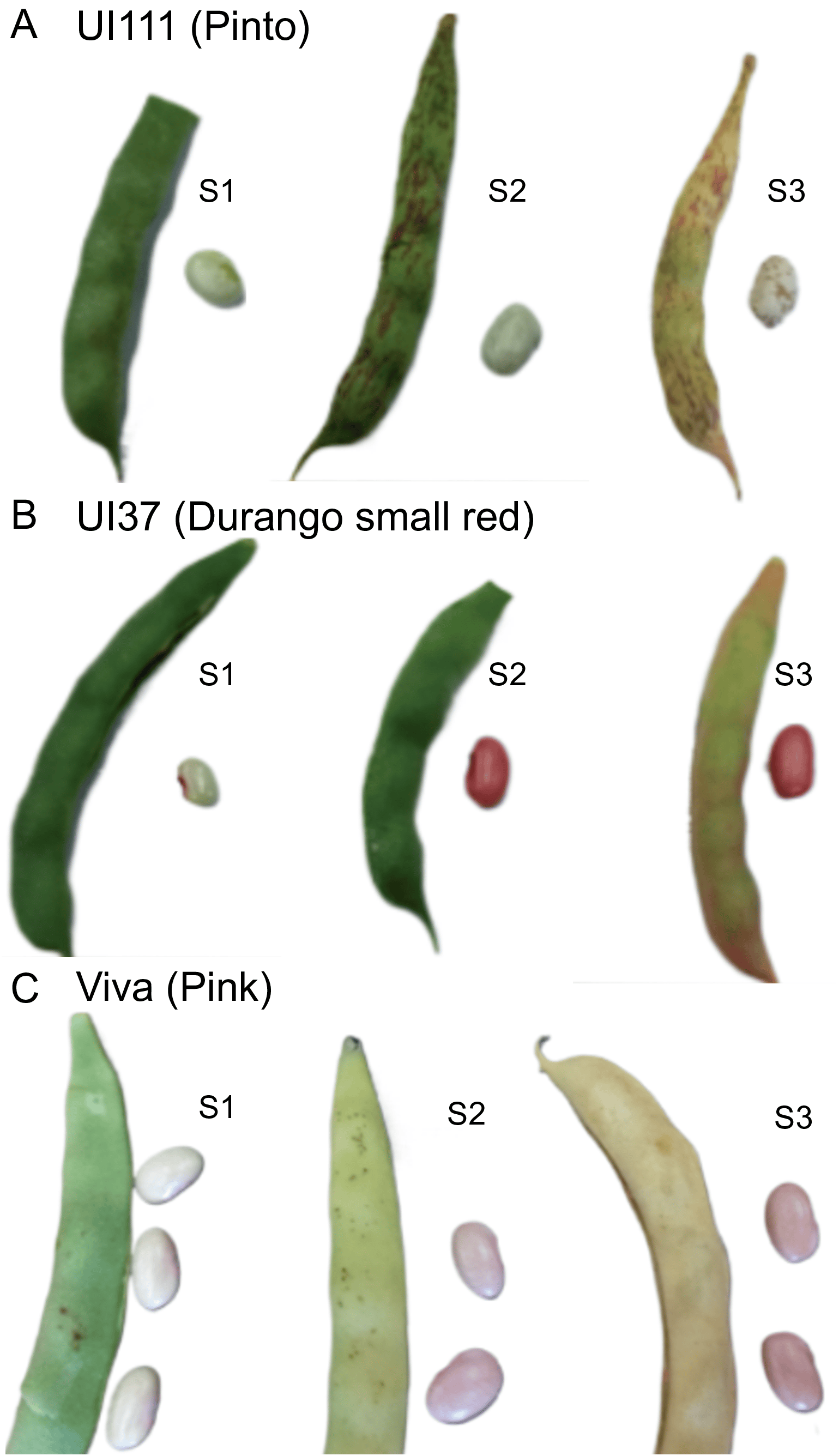

Supplement: Supplementary file 1 — Supplementary Material 1: Seed developmental stages and seed coat pigment acquisition of collected samples at three different stages (S1, S2, S3) for RT-qPCR analysis. A) samples of UI111 genotype consisted of green seeds with no stripe (S1), green seeds with stripe (S2), yellow seeds with stripe (S3); B) UI37 genotype comprised of green immature seeds (S1), red color started to develop on the seed coat (S2), and red color retained completely on the seed coat (S3); C) Viva genotypes with no color on seeds, mostly white (S1), pink color started to develop on the seed coat (S2), seeds retained complete pink color (S3) [file 12870_2025_6282_MOESM1_ESM.png]

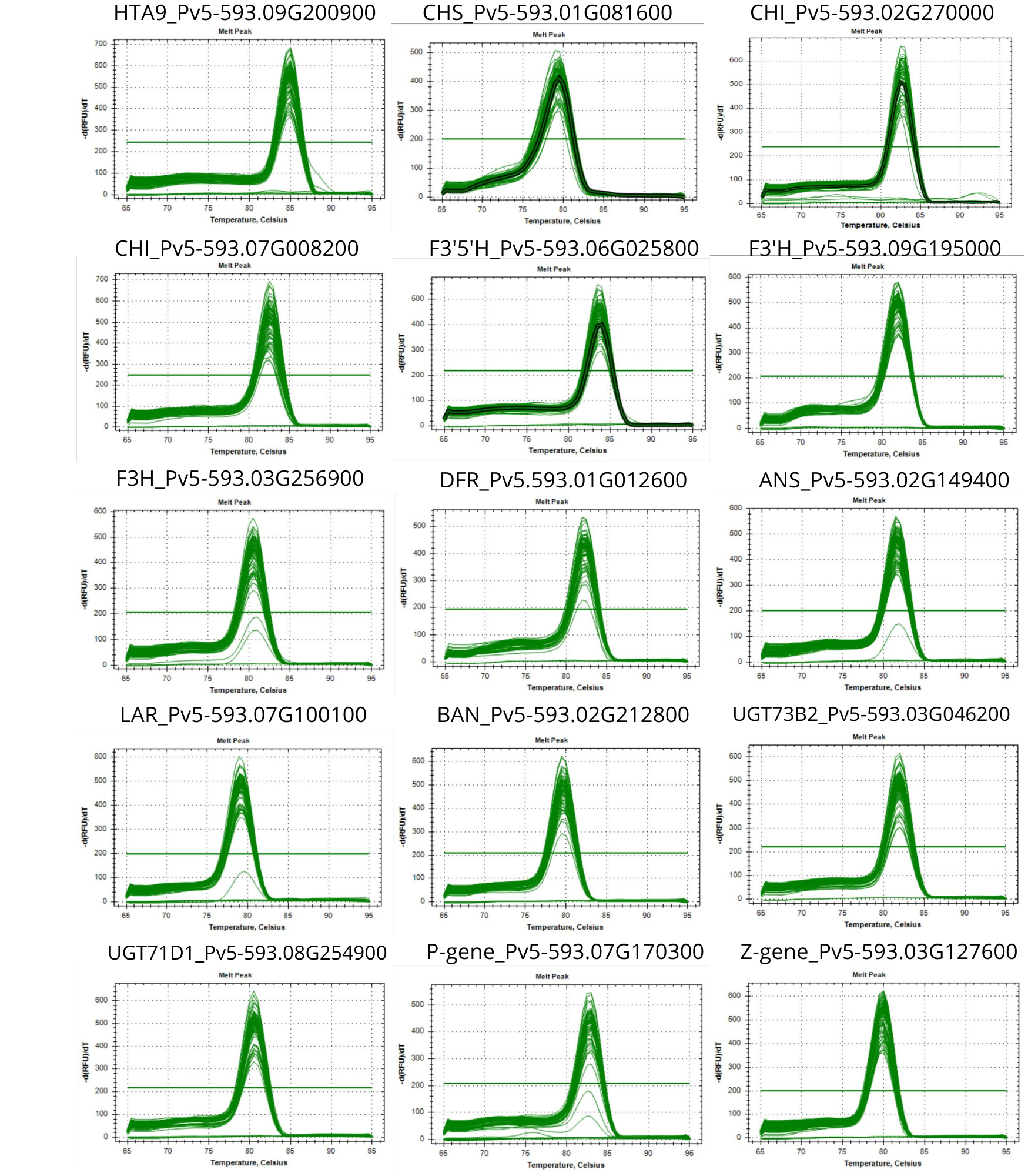

Supplement: Supplementary file 2 — Supplementary Material 2: Melt curve analysis to determine the gene specific primers to be used in RT-qPCR analysis. Single peak in the melt curve plot indicates the deigned primer is gene specific [file 12870_2025_6282_MOESM2_ESM.png]

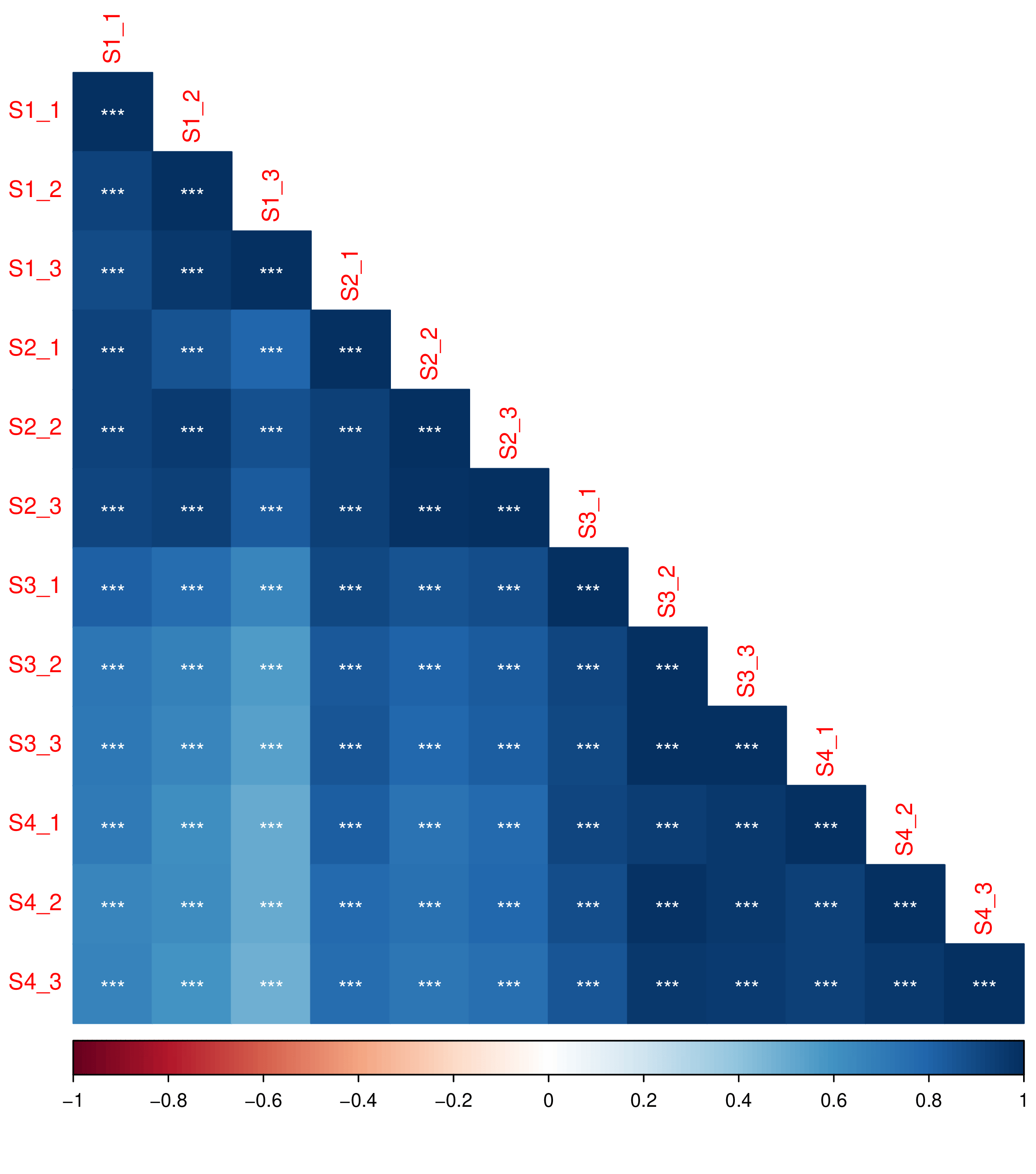

Supplement: Supplementary file 3 — Supplementary Material 3: Correlation heatmap between the biological replicates of different seed coat stages tissues developed based on the raw reads from RNA-Seq data. S1, S2, S3, and S4 represent the RNA sample collected from seed coat stage 1, 2, 3, and 4 respectively and the numeric value after “_” sign refers to the assigned biological replicate. Blue and red colors indicate positive correlation and negative correlation, respectively [file 12870_2025_6282_MOESM3_ESM.png]

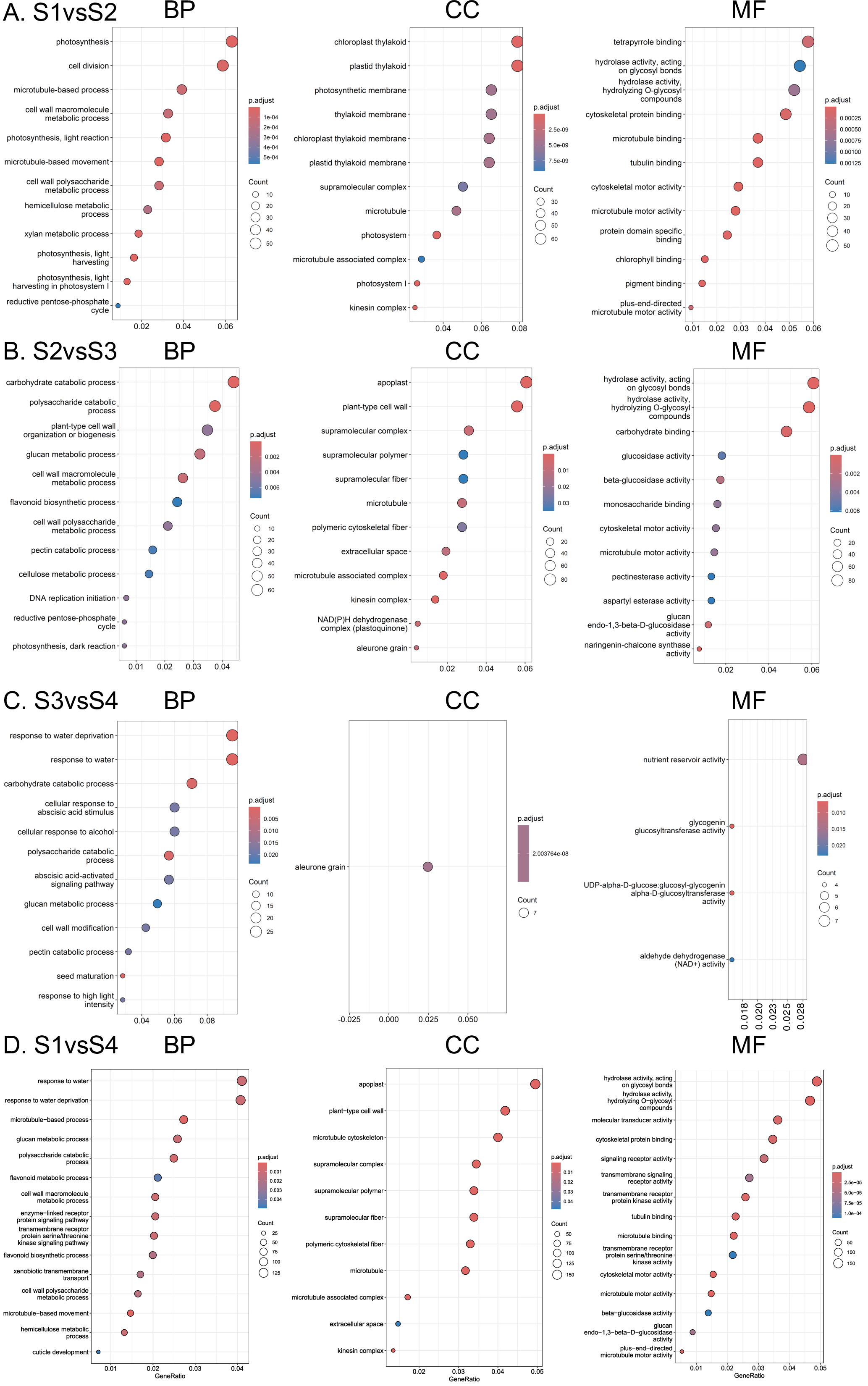

Supplement: Supplementary file 4 — Supplementary Material 4: Gene Ontology (GO) annotation and over-representation analysis of differentially expressed genes in five pairwise comparisons. Dot plots represent GO annotation of DEGs from A) S1 vs. S2, B) S2 vs. S3, C) S3 vs. S4, and D) S1 vs. S4 comparisons into three categories: biological processes (BP), cellular components (CC), and molecular functions (MF) [file 12870_2025_6282_MOESM4_ESM.png]

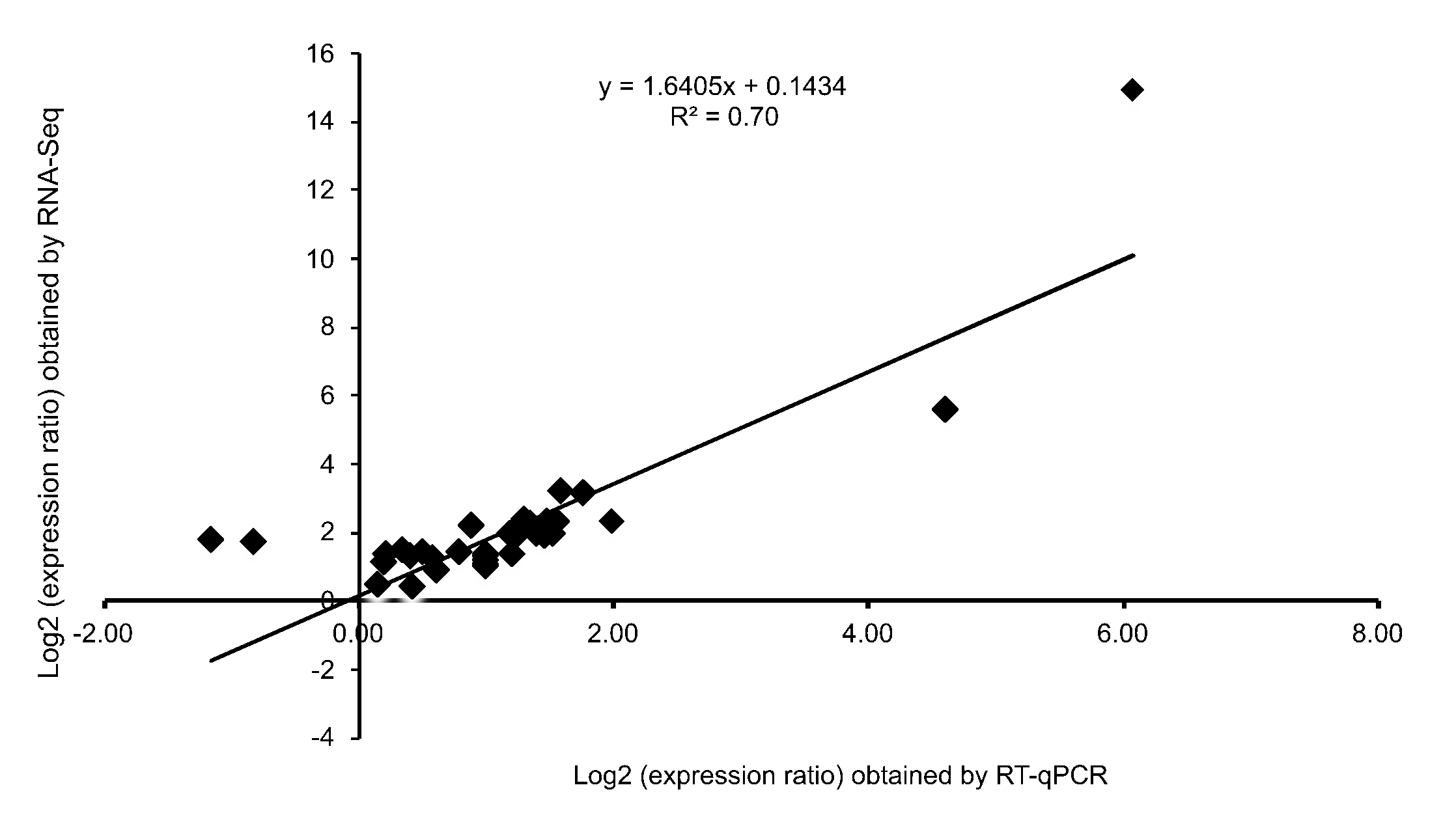

Supplement: Supplementary file 5 — Supplementary Material 5: Validation of 5-593 RNA-Seq data by RT-qPCR through the comparison of the gene expression ratios from qRT-PCR and RNA-Seq data. The correlation between RNA-Seq and RT-qPCR of the fold change in expression levels of the 14 genes in 5-593 [file 12870_2025_6282_MOESM5_ESM.png]

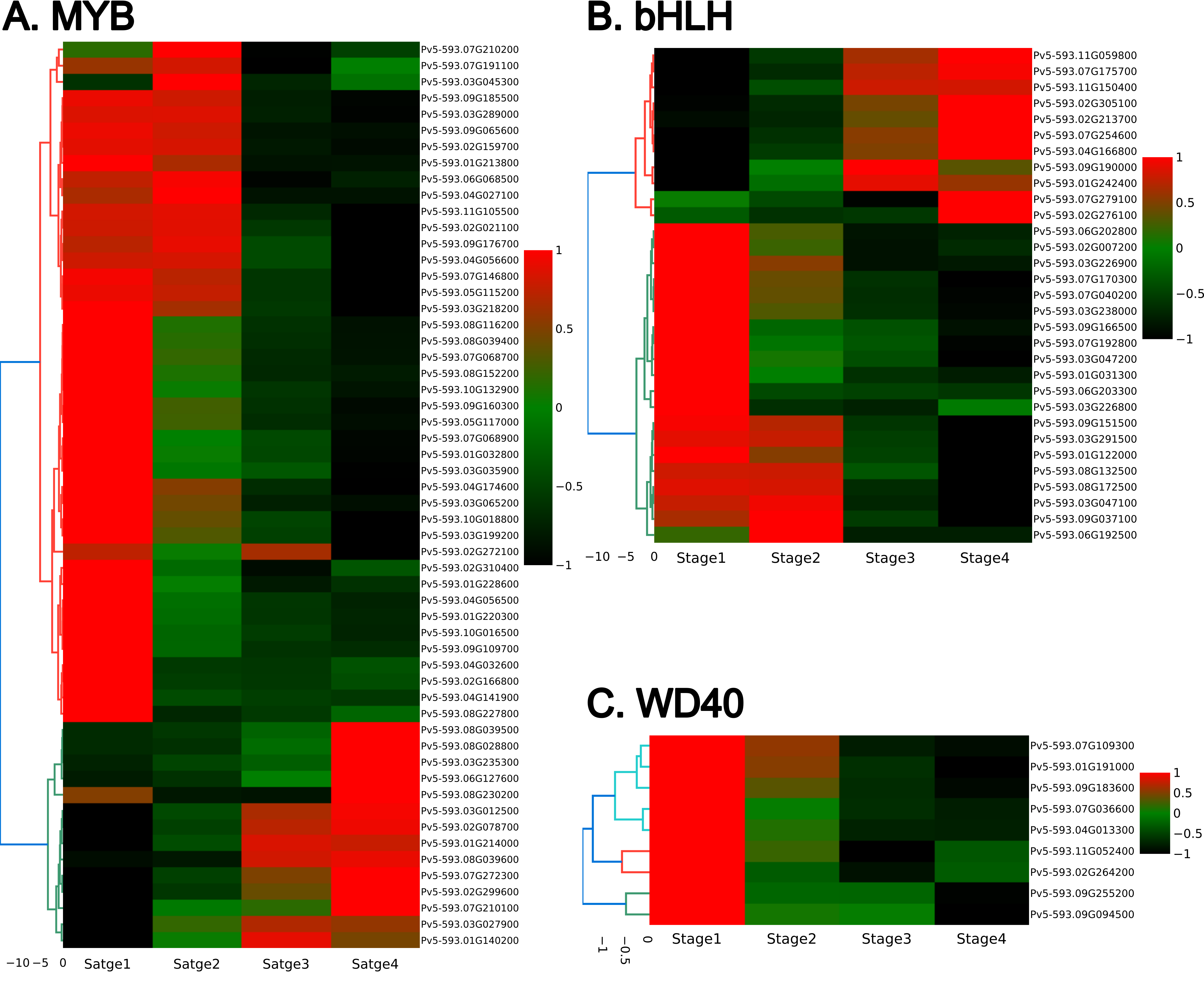

Supplement: Supplementary file 6 — Supplementary Material 6: The expression profiles of MYB-bHLH-WD40 DEGs during seed coat pigment development. (A) 56 MYB family genes; (B) 31 bHLH family genes; and (C) 9 WD40 family genes. Gene expression was scaled in this analysis using TPM Z-scores based on the mean value of three biological replicates in the heatmap. The key is located on the right-hand side in each case with TPM values increasing from black, green to red [file 12870_2025_6282_MOESM6_ESM.png]
