# Supplementary material for: Seed coat transcriptomic profiling of 5-593, a genotype important for genetic studies of seed coat color and patterning in common bean (Phaseolus vulgaris L.)
Source: BMC Plant Biol. 2025 Mar 5;25:284. doi: 10.1186/s12870-025-06282-7 (PMC11881399; doi:10.1186/s12870-025-06282-7)
Supplement: Supplementary file 7 — Supplementary Material 7: Scripts and pseudocode for RNA-Seq data preprocessing, aligning, and counting reads [file 12870_2025_6282_MOESM7_ESM.docx]

**Table S1.** Scripts and pseudocode for RNA-Seq data preprocessing, aligning and counting reads

##################################################################################################

## Pseudocode for RNA-Seq data preprocessing, aligning and counting reads

##################################################################################################

PATH = /PATH_TO_DIRECTORY/ (reference genome / output directory)

XXXX = FILE_PREFIX (e.g., LIBID, library ID)

FILE = genome/gff3 fasta file prefix

NCORES = 6 (number of threads)

# = * = * = * = * = * = * = * = * = * = * = * = * = * = * = *

### 1. Preprocess raw RNA-seq fastq files and QC (aka Prep reads)

# = * = * = * = * = * = * = * = * = * = * = * = * = * = * = *

## https://github.com/OpenGene/fastp

## -- gzipped format raw fastqs

fastp --in1 PATH/XXXX.R1.fastq.gz --in2 PATH/XXXX.R2.fastq.gz --out1 PATH/XXXX.R1.trimmed.fastq.gz --out2 PATH/XXXX.R2.trimmed.fastq.gz -l 50 -h XXXX.html &> XXXX.log

## -- bzipped format raw fastqs

fastp --in1 <(bzcat PATH/XXXX.R1.fastq.bz2) --in2 <(bzcat PATH/XXXX.R2.fastq.bz2) --out1 PATH/XXXX.R1.trimmed.fastq.gz --out2 PATH/XXXX.R2.trimmed.fastq.gz -l 50 -h XXXX.html &> XXXX.log

## -- QC using fastqc

# https://www.bioinformatics.babraham.ac.uk/projects/fastqc/

fastqc --threads NCORES -o PATH/fastqc PATH/*.fastq.gz

# = * = * = * = * = * = * = * = * = * = * = * = * = * = * = *

### 2. Align prepped reads using STAR aligner ##

# = * = * = * = * = * = * = * = * = * = * = * = * = * = * = *

## https://github.com/alexdobin/STAR

# = * = * = * = * = * = * = * = * = *

## -- 2a. Generate GTF file (i.e., convert gene_exons.gff3 to gene_exons.gtf)

# = * = * = * = * = * = * = * = * = *

gffread FILE.gff3 -T -o FILE.gtf

# = * = * = * = * = * = * = * = * = *

## -- 2b. Generate genome index using STAR (ONLY ONCE PER GENOME.VERSION

# = * = * = * = * = * = * = * = * = *

STAR --runMode genomeGenerate --runThreadN NCORES \

--genomeDir PATH/STAR_INDEX \

--genomeFastaFiles PATH/GENOME.FASTA \

--sjdbGTFfile PATH/FILE.gtf? \

--sjdbOverhang 100

## NOTE from START: If reads are of varying lengths you could use --sjdbOverhang is max(Read_length)-1.

## However, the default value of 100 works equally well.

STAR --runMode genomeGenerate --runThreadN NCORES --genomeDir PATH/STAR_INDEX --genomeFastaFiles PATH/GENOME.FASTA --sjdbGTFfile PATH/FILE.gtf --sjdbOverhang 100

# = * = * = * = * = * = * = * = * = *

## -- 2c. Align reads

# = * = * = * = * = * = * = * = * = *

STAR --genomeDir PATH/STAR_INDEX \

--runMode alignReads \

--runThreadN NCORES \

-c \

--readFilesIn PATH/XXXX.R1.fastq.gz PATH/XXXX.R2.fastq.gz \

--readFilesCommand gunzip \

--outFileNamePrefix PATH/XXXX

--outSAMtype BAM SortedByCoordinate \

--outFilterMultimapNmax 7 \

--outFilterMismatchNmax 4 \

--outSAMstrandField intronMotif \

--quantMode GeneCounts \

# = * = * = * = * = * = * = * = * = *

## -- 3. Count reads aligned to genes using featureCounts in Subread

# = * = * = * = * = * = * = * = * = *

## https://subread.sourceforge.net/SubreadUsersGuide.pdf

featureCounts -F GTF -p -s 2 -T nTHREADS NCORES --countReadPairs -t exon -g gene_id -a PATH/FILE.GTF -o XXXX.counts.txt PATH/XXXX.bam
